# Combining Morphology and Genetics in Resolving Taxonomy–A Systematic Revision of Spined Loaches (Genus *Cobitis*; Cypriniformes, Actinopterygii) in the Adriatic Watershed

**DOI:** 10.1371/journal.pone.0099833

**Published:** 2014-06-11

**Authors:** Ivana Buj, Radek Šanda, Zoran Marčić, Marko Ćaleta, Milorad Mrakovčić

**Affiliations:** 1 Department of Zoology, Faculty of Science, University of Zagreb, Zagreb, Croatia; 2 National Museum, Prague, Czech Republic; 3 Faculty of Teacher Education, University of Zagreb, Zagreb, Croatia; Laboratoire de Biologie du Développement de Villefranche-sur-Mer, France

## Abstract

Taxonomic investigation of spined loaches from Dalmatia and Herzegovina was conducted on specimens from 14 localities. The results of the detailed morphological investigations were combined with genetic data (based on one mitochondrial and two nuclear genes) in order to resolve the taxonomic status of each *Cobitis* population. Among the investigated features of external morphology, the appearance of spots on the caudal fin base turned out to have the greatest diagnostic value. Furthermore, the number of branched fin rays enabled the discrimination of several species. No morphometric character alone could ensure determination of any *Cobitis* species. Nevertheless, groups of populations that are more similar in their body shapes correspond to mitochondrial phylogenetic lineages. Based on molecular genetic markers, Dalmatian and Herzegovinian spined loaches form independent lineages inside the Adriatic phylogenetic group. Mitochondrial DNA phylogenetic reconstruction revealed six monophyletic lineages, corresponding to six species distributed in the investigated area. The population distributed in Mostarsko blato karstic field in Bosnia and Herzegovina is described as a new species based on a unique combination of morphological characters: a single triangular Canestrini scale; usually 5^1^/_2_ branched anal fin rays, 6^1^/_2_ branched dorsal fin rays, 14 branched caudal fin rays; no spots in the surface pigmentation layer on the caudal fin base; scales on the body very small.

## Introduction

Recognition and delimitation of species, the main objectives of systematics, are of crucial importance for implementation of adequate strategies for biodiversity conservation. Exclusive reliance on morphological characters, that were traditionally used to identify species, turned out to underestimate diversity by failing to detect cryptic taxa [1]. Genetic data are frequently used to delimit species, when conclusions about species status are based on an exclusivity criterion, such as reciprocal monophyly or degree of genetic clustering [2], [3]. However, those methods are also subjective and fail to account for several phenomena that cause gene tree incongruence with species tree [1]. Finally, recent implementations of coalescent model-based approaches allow conduction of probabilistic tests of species limits [4], [5]. Although some authors consider that integrative methods will not yield a clear result in cases when cryptic species are present [1], the critical review of the results of all mentioned methods may have the greatest resolution power in delimiting species, especially in cases when phenomena such as hybridization are more widespread, species are morphologically very similar and/or divergence happened more recently. Among such, “more difficult” taxonomic cases are freshwater spined loaches.

Even though allopatric speciation has been highlighted as responsible for divergence of many freshwater species, there are evidences of sympatric/parapatric speciation modes [6], [7], as well as contribution of genetic mechanisms other than selection (i.e. drift and founder events) [6] in freshwater fish studies. Fishes in general present a very diverse array of speciation scenarios (reviewed in [8]).

The genus *Cobitis* (spined loaches) comprises about 60 species of freshwater fishes, [9], [10], [11] that are distributed throughout the temperate zone of Europe and Asia. Due to the morphological similarity of different *Cobitis* species, and the existence of sibling species [12], their taxonomic differentiation is complicated and their systematics not completely resolved. Sibling species are defined as a cryptic sister species; two species that are the closest relatives and have not been distinguished from one another taxonomically [13]. Recent descriptions of new species and the discovery of significant diversity among spined loaches has been made possible by a combination of detailed morphological investigations (e.g. [11], [12], [14], [15]), karyological analyses (e.g. [16], [17]) and analysis of DNA markers (e.g. [18]–[22]).

**Figure 1:**
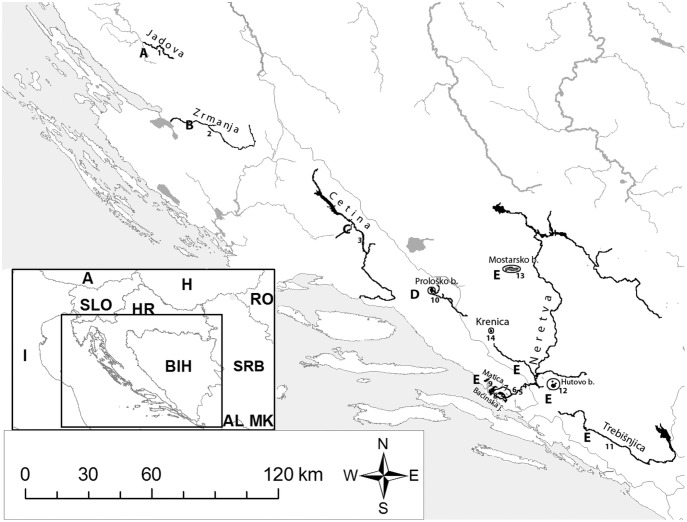
Map of investigated area with sampling localities marked, as well as distribution of *Cobitis* species based on literature. Legend: A = *C. jadovaensis*; B = *C. bilineata*; C = *C. dalmatina*; D = *C. illyrica*; E = *C. narentana*; 1 = Jadova; 2 = Zrmanja (HE Velebit accumulation); 3 = Cetina (Blato); 4 = Neretva in Metković; 5 = Mislina; 6 = Norin; 7 = Modro oko; 8 = Baćinska Lakes; 9 = Matica; 10 = Prološko blato; 11 = Trebišnjica; 12 = Hutovo blato; 13 = Mostarsko blato; 14 = Krenica; A = Austria; H = Hungary; SLO = Slovenia; HR = Croatia; I = Italy; BIH = Bosnia and Herzegovina; RO = Romania; SRB = Serbia; MK = The former Yugoslav Republic of Macedonia.

Dalmatia, which includes rivers in the Adriatic watershed in Croatia and Bosnia and Herzegovina, is well known for its high degree of endemism of freshwater fishes [23]–[25]. Several molecular genetic investigations of spined loaches in this region [18], [21], [22] have revealed that it is inhabited by *Cobitis* populations belonging to the Adriatic phylogenetic group, a distinct monophyletic group. It is currently thought that the Adriatic watershed in Croatia is inhabited by five different *Cobitis* species (Figure 1): *C. jadovaensis* Mustafić & Mrakovčić, 2008, a recently described species living only in the small karstic river Jadova [11]; *C. bilineata* Canestrini, 1865, which inhabits the Zrmanja River in Croatia [21], [22], but is also distributed in Slovenia, Italy, France, Switzerland and Spain [9]; *C. dalmatina* Karaman, 1928, endemic to the Cetina River in Croatia [26], [14]; *C. illyrica* Freyhof & Stelbrink, 2007, another recently described species with its only known locality being Prološko blato, a small lake in the Imotsko Polje karstic field [15]; and *C. narentana* Karaman, 1928, supposedly distributed in basin of the Neretva River, namely in the lower courses and tributaries of the Neretva River, as well as in the Baćinska Lakes and Matica River [27]. Recent descriptions of two species from southern Croatia (*C. jadovaensis* and *C. illyrica*) were based on morphological characters [11], [15]. In the Adriatic watershed in Bosnia and Herzegovina spined loaches have been recorded in the Neretva basin (including Hutovo blato wetland and Mostarsko blato karstic field), in Trebišnjica River, and also in Krenica Lake, which is located in Bekijsko karstic field [28] (Figure 1). Whereas Šanda et al. [28] suggested the existence of another *Cobitis* species in addition to *C. narentana* in this area of Bosnia and Herzegovina, neither the taxonomic status of the populations nor the distribution of each species have been ascertained.

Although previous investigations recognized the region of Dalmatia and Herzegovina as an area inhabited by a large number of *Cobitis* species comprising many endemics, the diversity of spined loaches in the area is probably still underestimated, while data on intraspecific diversity being especially scarce. The greatest problem with the investigations performed so far is that all of them were based either on morphological characters or on DNA markers, and none on both, and usually on very few specimens from one or a few populations. This is especially true for the description of *C. illyrica* [15] where the sample size analyzed is too low to represent the morphological diversity and distribution of the species. Original descriptions of *C. dalmatina* and *C. narentana* [26] are also problematic, consisting of a few very general sentences in the original text. Other investigations gathered under one supposed taxon specimens that, based on today’s knowledge, belong to more than one species. For example, in the investigations of Schneider et al. [14], [27] in the sample of *C. narentana*, *C. illyrica* specimens were also included.

The aim of the present investigation was to determine the taxonomic status, phylogenetic relationships, level of intraspecific and intrapopulational diversity and the distribution of spined loaches in the region of Dalmatia and Herzegovina. Furthermore, based on large samples from more than one population (where possible) for each species, we tested the diagnostic markers traditionally used in *Cobitis* species determination and ascertained the diagnostic value of each morphological character.

## Materials and Methods

### Ethics Statement

This investigation was conducted entirely in accordance with ethical standards and Croatian legislation. The work was approved by the Ethical Committee of the Faculty of Science, University of Zagreb. The permission for sampling on all localities in Croatia was issued by the Ministry of Agriculture, Forestry and Water Management of the Croatia. The permission for sampling on Hutovo blato, Trebišnjica, Krenica and Mostarsko blato was issued by the Ministry of Agriculture of Bosnia and Herzegovina. The sampling was conducted by electrofishing. The fishes were over-anaesthetized with tricaine-methanesulphonate (MS 222) and preserved in ethanol. All specimens are deposited in institutional collections (see List of examined material).

Specimens were collected from 14 localities in Dalmatia and Herzegovina (List of examined material, Figure 1). All rivers and lakes where spined loaches have been recorded inside the investigated area were included in the study. Where possible, sampling was conducted at more than one locality in each river.

In order to ascertain the taxonomic position and phylogenetic relationships of the Adriatic spined loaches, two types of analyses were employed – the investigation of morphology and the analyses of molecular phylogeny. Morphological analyses comprised the analyses of morphometry, meristics and external morphology.

A total of 24 morphometric characters were measured using an electronic caliper to the nearest 0.01 mm: total length (TL), standard length(SL), lateral head length (c), preanus distance (Pan), preanal distance (aA), prepelvic (preventral) distance (aV), prepectoral distance (aP), predorsal distance (aD), caudal peduncle length (lpc), length of dorsal (lD), anal (lA), caudal (lC), pectoral (lP) and pelvic (ventral) (lV) fins, distance between pelvic fins and anal aperture (Van), head depth (hc), maximum body depth (H) and caudal peduncle depth (or minimum body depth, h), head width (laco), maximum body width (lac), distance between eyes (io), eye diameter (o), preorbital distance (prO), postorbital distance (poO). All measurements were made point to point by one author. Size-dependent variation was removed by allometric transformation of morphometric measures using the formula [29], [30].
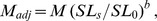
where M_adj_ is the adjusted size-independent measurement, M the original morphometric measurement, SL_s_ the overall mean of the standard length for all fishes from each population, SL_o_ the standard length of the fish, and b the slope of the regression of log M on log SL_o_ based on all specimens from each population. Correlation coefficients between transformed, adjusted measurements and standard length were calculated to confirm that the effect of size was removed.

Morphometric ratios, i.e. percentage ratios of morphometric characters in relation to SL, c and H, as well as h/lpc were also calculated in order to enable comparison with data in the literature, especially in cases when certain morphometric ratios were used to separate species.

To compare morphometric features between males and females, Student’s t-test was employed. Statistical comparison among populations was conducted using analysis of variance (ANOVA) and principal component analysis (PCA), and was based on size-independent measurements. Software package STATISTICA 7.1 was used for data analysis.

The meristic characters assessed included the number of unbranched and branched fin rays in the dorsal, anal, pelvic, pectoral and caudal fins. The last two branched rays in dorsal and anal fins, which articulate on a single pterygiophore, were scored as ″1^1^/_2_″.

The external morphology was examined in detail in order to detect differences between populations and species and to reveal phylogenetic signals (defined as the degree to which phylogenetic relatedness among taxa is associated with their phenotypic similarity [31]) in their morphology. Attention was paid to characters traditionally used in *Cobitis* species differentiation, including the appearance of spots on the caudal fin base (see [9], [11], [14], [15], [32]), the appearance of Gambetta zones on body sides (see [9], [12]), structure of the scales (see [12]) and the position and shape of the suborbital spine (see [14], [32]). Gambetta zones [33] are pigmentation zones comprised of various spots and blotches. Loaches of the genus *Cobitis* have four Gambetta zones located along the body [9], usually recorded as Z1–Z4, zone Z1 being located dorsolaterally and Z4 ventrolaterally. Saitoh & Aizawa [34] reported that zone Z4 consists of two pigmentation layers; surface layer located in the dermis and deeper layer in the horizontal septum and its surrounding tissue. Likewise, pigmentation located on the beginning of the caudal fin is also formed by two pigmentation layers [34]; surface layer in the dermal tissue and the deeper layer in the connective tissue surrounding the base of fin rays. In this investigation we differentiated two layers of pigmentation, unlike majority of authors reporting morphological characters of spined loaches (e.g. [11] [14] [15]). Thereafter, our results may not be completely comparable with others due to the lack of information whether pigmentation patterns reported by other authors refer to the surface layer only or to the combination of both layers. The suborbital spine (*spinum suborbitale*) is a modified ethmoidal bone, situated below the eye.

An investigation of morphological features was conducted on 226 fishes collected from 13 sites (List of examined material) and preserved in ethanol. However, only a few males were sampled so a normal distribution could not be confirmed at the following localities: Jadova, Neretva in Metković, Norin and Prološko blato. Therefore, males from those locations were not included in the statistical comparative analyses. In Baćinska Lakes, despite intensive and widespread sampling and previous reports testifying to a large population of spined loaches (e.g. [27]), only two females were caught. Hence only molecular investigations were conducted on the specimens from these lakes.

Molecular analysis was undertaken on 122 specimens collected from 14 localities (List of examined material, Table 1) and was based on three molecular markers: mitochondrial cytochrome *b* (cyt *b*), nuclear RAG1 gene and the first intron of nuclear S7 gene.

**Table 1:** Sampling localities, haplotype codes and GenBank accession numbers of specimens included in the phylogenetic analyses.

locality	species	cyt *b* haplotypes	RAG1 haplotypes	S7 haplotypes	accession number	reference
Jadova	*C. jadovaensis*	JAD1	rJAD1,2	sJAD1–4	KJ487435, KJ487503, KJ487504, KJ487534- KJ487537	this study
Zrmanja	*C. bilineata*	ZRM1–5	rZRM1–3	sZRM1,2	KJ487450, KJ487464, KJ487467, KJ487468, KJ487471, KJ487517-KJ487519, KJ487553, KJ487554	this study
Cetina	*C. dalmatina*	CET1–12	rCET1–7	sCET1–7	EF605302-EF605306, KJ487457-KJ487461, KJ487463, KJ487470, KJ487497-KJ487503, KJ487527-KJ487533	this study
Mislina (Neretva)	*C. narentana*	NER1, 3, 8–11	rNER1–3, 5	sNER1–3, 5, 6	EF605315, EF605316, KJ487438- KJ487449, KJ487454-KJ487456, KJ487472, KJ487485, KJ487507-KJ487513, KJ487520, KJ487521, KJ487539-KJ487550	this study
Metković (Neretva)	*C. narentana*	NER5, 6, 7	rNER1	sNER1, 6, 10, 11		
Norin	*C. narentana*	NER2, 3, 6, 11, 12, 14, 16, 18, 19	rNER1, 4–6	sNER3, 12, 13, 14		
Modro oko	*C. narentana*	NER1, 3, 11–15, 17	rNER1, 2, 6–8	sNER1, 5, 7–9		
Trebišnjica	*C. narentana*	NER1, 7, 12, 13	rNER1	sNER1–4		
Hutovo b.	*C. narentana*	NER1, 4				
Baćinska Lakes	*C. illyrica*	BAC1	rMAT1, 2	sMAT1, sKRE4	EF605312, EF605313, KJ487436, KJ487437, KJ487451-KJ487453, KJ487465, KJ587466, KJ487469, KJ487479-KJ487484, KJ487486, KJ487487, KJ487489-KJ487496, KJ487560, KJ487514-KJ487516, KJ487522, KJ487524-KJ487526, KJ487538, KJ487551, KJ487552	this study
Matica	*C. illyrica*	MAT1–6	rMAT1–4	sMAT1, sKRE1, 4		
Krenica	*C. illyrica*	KRE1–6, MAT5	rKRE1–6, rMOB1	sKRE1–4		
Prološko b.	*C. illyrica*	PRO1–4	rPRO1–3, rKRE1–3, rMAT4	sPRO1, sKRE1		
Mostarsko blato	*C. herzegoviniensis* Buj & Šanda, sp. nov.	MOB1–6	rMOB1–2, rKRE1	sMOB1, sKRE1	KJ487473-KJ487478, KJ487486-KJ487488, KJ487522, KJ487523	this study
	*C. arachthosensis*	1			AY191581	[20]
		2			AF263088	[18]
	*C. bilineata*	1–10	rBIL2–5	sBIL1–3	EF672359-EF672368, EF672415- EF672418 EF672447-EF672449	[22]
		11–12			AF263090, AF263091	[18]
			rBIL1		EF056382	[37]
	*C. calderoni*	1, 2			AY860121, AY860122	[64]
	*C. elongata*	1–4			EF605308, EF605310, EF605318, EF605320	[21]
			rELO1		EF056332	[37]
			rELO2, 3	sELO1, 2	EF672420, EF672421	[22]
	*C. elongatoides*				AY191567	[20]
	*C. hellenica*	1, 2			AY191582, AY191583	[20]
	*C. melanoleuca*		rMEL1		CF056343	[37]
	*C. meridionalis*	1, 2			AF263083, AF263084	[18]
	*C. ohridana*	1–5			EF597224, EF597226, EF597227, EF597234, EF597240	[65]
		6, 7	rOHR1–3	sOHR1–3	EF672394, AY191563, EF672431- EF672433, EF672463-EF672465	[22]
	*C. paludica*	1, 2			AY860179, AY860180	[64]
	*C. punctilineata*	1, 2			AY191579, AY191580	[20]
	*C. stephanidisi*	1, 2			AY191571, AY191572	[20]
	*C. strumicae*	1, 2			DQ217372, DQ217373	[20]
	*C. taenia*	1			AY191565	[20]
		2			AF263078	[18]
			rTEN1		EF056334	[37]
	*C. tanaitica*	1, 2			DQ217397, DQ217398	[20]
	*C. trichonica*	1, 2			AF263085, AF263086	[18]
	*C. vardarensis*	1, 2			AY191569, AY191570	[20]
	*C. vettonica*	1, 2			AY860182, AY860183	[64]
	*C. zanandreai*	1–4	rZAN1, 2	sZAN1, 2	EF672411-, EF672413, AY191562, EF672444, EF672445, EF672477, EF672476	[22]

Total genomic DNA was extracted from fresh and deep-frozen fin tissue using a standard extraction product (DNeasy tissue kit, Qiagen). Polymerase chain reaction (PCR) amplifications were performed in a 50µl reaction volume containing 25µl of HotStarTaq Master Mix (Quiagen), 2µl of each primer and 4µl of template DNA. Cyt *b* gene was amplified using primers L14725 and H16460 [35] and the following temperature regime: 15 min at 95°C; 35 cycles of 30 s at 94°C, 30 s at 50°C and 90 s at 72°C; 7 min at 72°C. The primers and PCR protocols for amplification of RAG1 and S7 genes are described in [22], [36] and [37]. Sequencing was carried out by Macrogen Service Centre (Seoul, South Korea) with internal primers H-COB_cyt638 and L-Cyp_425 [21] for cyt *b*. The same primers as for the PCR were used for sequencing of RAG1 and S7 genes.

Homologous regions of cyt *b* and RAG1 genes were aligned manually against previously published sequences. Chromatograms and alignments were checked visually and were found to contain no gaps or stop codons. Because of the presence of insertions and deletions in the S7 gene, those sequences were aligned with Clustal X [38]. Haplotype variants of nuclear genes in heterozygous individuals were reconstructed by a Bayesian statistical method implemented in PHASE 2.1 software [39], [40]. The phase reconstruction method (described in details in [39]) regards the unknown haplotypes as unobserved random quantities and aims to evaluate their conditional distribution in light of the genotype data, using Gibbs sampling, a type of Markov Chain Monte Carlo algorithm. The analyses were run five times for both nuclear genes with different values of the seed of the pseudo-random number generator. Each run consisted of a burn-in-period of 100 followed by 1000 iterations. The gametic phase estimation was consistent through the runs according to goodness-of-fit measures. Nucleotide composition was examined by the χ^2^ homogeneity test of base frequencies implemented in PAUP (version 4.0b10 [41]) for each data set, as well as the transition/transversion ratio (ts/tv). In order to test whether all mutations were selectively neutral, statistical tests D* and F* (proposed by Fu & Li [42]) and that of Tajima [43] were conducted using DnaSP v5 [44]. The same software was employed to estimate the recombination parameter, R [45] and the minimum number of recombination events, RM [46], for each data set. For phylogenetic analyses, in addition to the sequences obtained in the present study, sequences available on GenBank were also included (Table 1).

Pairwise comparisons of uncorrected sequence divergence (p-distances) of cyt *b* and RAG1 were analyzed using MEGA version 3.1 [47]. P-distances were not calculated for the S7 first intron because the presence of insertions and deletions of significant length (up to 11 base pairs) would lead to overestimates.

In order to ascertain the phylogenetic position of Dalmatian and Herzegovinian spined loaches within the phylogenetic tree of European *Cobitis*, and to resolve the phylogenetic relationships among them, three different methods of phylogenetic reconstruction were employed: maximum likelihood (ML), maximum parsimony (MP), both implemented in PAUP, and Bayesian inference (BAY), implemented in MrBayes (version 3.1.2 [48]). ML analysis was performed under the heuristic search option using the tree bisection-reconnection (TBR) branch swapping algorithm. For MP analysis, a heuristic search mode with 100 replicates was used, with randomized input orders of taxa, and TBR branch swapping with all codon sites and nucleotide substitutions types weighted equally. Nonparametric bootstrapping (100 pseudo-replicates for ML, 1000 for MP, 10 additional sequence replicates) was used to assess branch support (BS). Each BAY analysis consisted of two simultaneous runs. For each, Markov Chain Monte Carlo was run four times for three million generations with trees sampled every 100 generations. The first 20% of the sampled trees were discarded and Bayesian posterior probabilities (BPP) were estimated from the 50% majority-rule consensus tree of the retained trees. For ML and BAY analyses, the best-fitting model of molecular evolution was selected by hierarchical likelihood ratio tests using MODELTEST software (version 3.06 [49]). Phylogenetic analysis was performed on each investigated genetic marker independently, due to differences in data sets composition (depending on the available sequences in GenBank) and differences in the mutational rate and phylogenetic performance of each investigated gene. Sequences of *Sabanejewia romanica* (Baçescu, 1943) [18], which belongs to the same family as *Cobitis*, and *Cyprinus carpio* L., 1758, from a different family though the same order, were used as outgroups for cyt *b* phylogenetic reconstruction. For the RAG1 data set, a sequence of *S. balcanica* was used for rooting, while for S7, *C. elongatoides* and *C. strumicae* served as outgroups.

In order to incorporate the possibility of horizontal gene transfer into the phylogenetic reconstruction of nuclear DNA, phylogenetic networks of nuclear haplotypes were constructed based on a median-joining (MJ) algorithm using Network 4.5.1.6. software (Fluxus Technology Ltd.).

To undertake a detailed phylogenetic reconstruction of spined loaches from Matica R., Prološko blato, Baćinska lakes, Krenica and Mostarsko blato, and to confirm their taxonomic status, cyt *b* sequences from those localities were analyzed by a statistical parsimony method [50] under a 95% connection limit, using the program TCS (version 1.21 [51]).

As a complementation to already described morphological and phylogenetic analyses, we have also employed Bayes factors (BF) for comparison of phylogenetic hypotheses or models. That approach is one of recently described coalescent-based methods for statistical species delimitation using multi-locus DNA sequence data [5]. BF is calculated as the ratio of the marginal likelihoods of two models, which has the advantage of taking into account priors used in Bayesian analyses [52]. Even though reliance on Bayes factors as a species delimitation model tool has not been thoroughly explored [5], available examples approve their usefulness in model averaging [1], [5]. In this investigation we have used BF to test six phylogenetic models (scenarios) that were created based on the results of all other analyses. Model A corresponds to our conclusion that each mtDNA sublineage represents separate species (six species); in model B seven species were included because *C. bilineata* was considered as two species. Model C comprised six species, because *C. illyrica* and *C. herzegoviniensis* Buj & Šanda, sp. nov. were considered as one species; whereas for model D *C. dalmatina* and *C. narentana* are also considered as one species. Model E relies on the RAG1 gene tree so it comprises four species (*C. jadovaensis*, *C. narentana* + *C. bilineata*, *C. dalmatina*, *C. illyrica + C. herzegoviniensis* Buj & Šanda, sp. nov.). Likewise, model F is based on the S7 first intron so it contains only three species (*C. narentana* + *C. bilineata*, *C. dalmatina*, *C. illyrica* + *C. herzegoviniensis* Buj & Šanda, sp. nov. + *C. jadovaensis*). We used the coalescent-based species tree program *BEAST [53] and prepared multi-locus data set (comprising sequences of three genes) with three partitions. Analyses were performed under an uncorrelated lognormal relaxed molecular clock, whereas a Yule process was used for the species tree prior. Analyses were run for one million generations with 20% of the trees discarded as burn-in. Marginal likelihood scores, as well as Bayes factors were calculated in Tracer v1.5. [54]. Besides the harmonic mean estimator (HME), we have also employed path sampling (PS) and stepping-stone sampling (SS) for estimation of the marginal likelihood values of competing models. Namely, previous investigations demonstrated better performance of recently developed techniques PS and SS than HME by not overestimating the true marginal likelihoods [1] [5]. PS explores an almost continuous progression of distributions along a path from the posterior to the prior when calculating the marginal likelihood [55], whereas stepping-stone sampling bridges the posterior and prior distributions [52] [5]. For PS and SS calculations appropriate code [1] was added into XML files generated by BEAUti v1.7.4 [56].

Finally, nucleotide sites that represent fixed differences between *Cobitis* species (as proposed in this investigation), as well as shared polymorphisms, were detected using SITES [57].

### List of Examined Material

Institutional abbreviations: NMP, National Museum, Prague; PMF, Faculty of Science, Zagreb; PMR, Natural History Museum, Rijeka.

*Cobitis jadovaensis*: PMF CBJA1–6, 6 spec. (morphological analyses: PMF CBJA1–6: 2♂, 4♀; genetic analyses: PMF CBJA 1–6), SL 60.8–87.0 mm, Jadova R., Croatia.

*Cobitis bilineata*: PMF CBZR6–32, 27 spec. (morphological analyses: PMF CBZR7–32: 7♂, 19♀; genetic analyses: PMF CBZR6–12, 14, 15, 16, 19, 20, 28), SL 54.0–88.0 mm, Zrmanja R., Croatia.

*Cobitis dalmatina*: PMF CBCE1–15, PMF CBCE 18–20, PMF CBCE 22–26, 23 spec. (morphological analyses: PMF CBCE6–15, 18, 19, 20, 22–26: 11♂, 7♀; genetic analyses: PMF CBCE1–5, 7–9, 12–15, 23), SL 55.8–105.3 mm, Cetina R. (in Blato village), Croatia.

*Cobitis narentana*: PMF CBMI1–9, 9 spec: 4♂, 5♀ (all for morphological and genetic analyses), SL 56.8–88.3 mm, Neretva R. in Mlinište village (channel Mislina), Croatia; PMF CBNO 1–19, 19 spec. (morphological analyses: PMF CBNO1, 3–6, 9–19: 2♂, 15♀; genetic analyses: PMF CBNO1–7, 9–13, 15), SL 70.8–92.3 mm, Norin R., Croatia; PMF CBMO1–19, 19 spec. (morphological analyses: PMF CBMO 1–19: 4♂, 15♀; genetic analyses: PMF CBMO 1–4, 6–12, 14, 15, 18), SL 53.9–77.9 mm, Modro oko Lake, Croatia; PMF CBNE 5, 6, 7, 12, 13, 14, 6 spec. (morphological analyses: PMF CBNE6, 7, 12–14: 1♂, 4♀; genetic analyses: PMF CBNE5–7), SL 63.4–85.0 mm, Neretva R. in Metković, Croatia; NMP P6V 81060–81072, 81224–81239, 81699–81704, 37 spec. (morphological analyses: NMP P6V 81060–81072, 81224–81239: 14♂, 15♀; genetic analyses: NMP P6V 81699–81704), SL 50.9–74.6 mm, Trebišnjica R. (in Ravno, Popovo karstic field), Bosnia and Herzegovina; NMP P6V 81209–81223, 81928–81930, 18 spec. (morphological analyses: NMP P6V 81209–81223: 4♂, 11♀; genetic analyses: NMP P6V 81928–81930), SL 49.1–90.8 mm, Hutovo blato, Bosnia and Herzegovina.

*Cobitis illyrica*: PMF CBPR 1, 2, 4–25, 28, 29, 31, 27 spec. (morphological analyses: PMF CBPR7–25: 1♂, 18♀; genetic analyses: PMF CBPR1, 2, 4–6, 28, 29, 31), SL 52.7–98.7 mm, Prološko blato Lake (Imotsko polje karstic field), Croatia; PMF CBBA1, 2*, 2 spec. (used only for genetic analyses), SL 58.2–60.4 mm, Baćinska Lakes, Croatia; PMF CBMA 1–25*, 25 spec. (morphological analyses: PMF CBMA 6–25: 7♂, 13♀; genetic analyses: PMF CBMA1–5, 10, 14–17), SL 54.0–81.9 mm, Matica R. (Polje Jezero karstic field), Croatia; NMP P6V 81191–81208, 81986–81995*, 28 spec. (morphological analyses: NMP P6V 81191–81208: 9♂, 9♀; genetic analyses: NMP P6V 81986–81995), SL 59.2–77.6 mm, Krenica Lake (Bekijsko karstic field), Bosnia and Herzegovina;

*Cobitis herzegoviniensis:* Buj & Šanda, sp. nov: PMR VP 2950–52, NMP P6V 80903–80905, 81171–81190, 81959–81961, 81964, 81967–81970, 81972–81975**, 38 spec. (morphological analyses: PMR VP 2950–52, NMP P6V 80903–80905, 81171–81190: 18♂, 8♀; genetic analyses: NMP P6V 81959–81961, 81964, 81967–81970, 81972–81975), SL 51.2–73.0 mm, Mostarsko blato karstic field, Bosnia and Herzegovina.

*Based on literature data, those samples were regarded as *Cobitis narentana.*

**Based on literature data, those samples were considered as *Cobitis narentana.*

### Nomenclatural Acts

The electronic edition of this article conforms to the requirements of the amended International Code of Zoological Nomenclature, and hence the new names contained herein are available under that Code from the electronic edition of this article. This published work and the nomenclatural acts it contains have been registered in ZooBank, the online registration system for the ICZN. The ZooBank LSIDs (Life Science Identifiers) can be resolved and the associated information viewed through any standard web browser by appending the LSID to the prefix “http://zoobank.org/”. The LSID for this publication is: urn:lsid:zoobank.org:pub: 261362F1–0C5A-4491–9D4A–F600075AC6BC. The electronic edition of this work was published in a journal with an ISSN, and has been archived and is available from the following digital repositories: PubMed Central, LOCKSS.

## Results

The aim of the present investigation was to confirm the taxonomic status of all Dalmatian and Herzegovinian *Cobitis* populations. Therefore, the results were analyzed without taxonomic priors and are presented for each population. The current taxonomic status of each population according to the literature is presented in the Introduction and in Figure 1, while taxonomic conclusions based on our results are reported in the Discussion and in the other figures.

### Morphometric Characters

Morphometric analysis was conducted separately for females and males since statistically significant differences were found between the sexes, in both measured and transformed (size-independent) morphometric characters (t-test; p<0.05). Mean, minimal and maximal values of morphometric ratios for each population are reported in the Table S1.

All size-independent morphometric characters were significantly different among both females and males from different populations (ANOVA; in all cases α = 0.00). However, a *post hoc* comparison (Fisher’s LSD test) revealed that some groups of populations are more uniform regarding their body shape than others. Females from Krenica, Matica (considered as *C. narentana*) and Prološko blato (*C. illyrica*) possessed pectoral fins of uniform length, while this character was significantly different between those populations and the Mostarsko blato population (also supposedly belonging to *C. narentana*). Furthermore, males from Krenica and Matica were uniform with regard to lD, lA, lV and lac, whilst those measures were significantly different between those males and those from Mostarsko blato.

**Figure 2:**
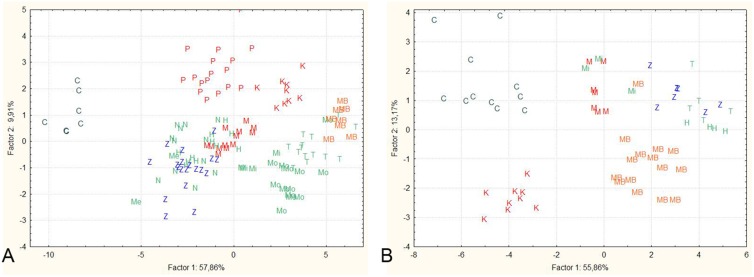
Plot of scores for factors 1 and 2 based on size-independent measures for females (A) and males (B) from all investigated populations. Legend: Z = Zrmanja (*C. bilineata*); C = Cetina (*C. dalmatina*); Me = Neretva in Metković (*C. narentana*); Mi = Mislina (*C. narentana*); N = Norin (*C. narentana*); Mo = Modro oko (*C. narentana*); H = Hutovo blato (*C. narentana*); T = Trebišnjica (*C. narentana*); M = Matica (*C. illyrica*); P = Prološko blato (*C. illyrica*); K = Krenica (*C. illyrica*); MB = Mostarsko blato (*C. herzegoviniensis* Buj & Šanda, sp. nov.).

The results of PCA analysis are shown on Figure 2. For females the first factor comprised 57.7% of the total variability and was mainly influenced by c, pan, pA, pV and pD, while the second factor accounted for 9.9% of the total variability and is mainly correlated with lpc, lC, h and io. Similarly, in the case of males, the first factor encompassed 55.9% of the total variability and mainly corresponds with c, pan, pA, pV, pD and prO, while the second factor, which comprises 13.2% of the total variance, is mainly influenced by lC, h, io and Oh. Projection of size-independent measures of females revealed that the Cetina population is, based on standardized morphometric characters, different from all the other populations. Separation of populations from Prološko blato, Matica and Krenica, as well as the one from Mostarsko blato, was also obvious. Each of these populations was, based on body shape, separated into its own group. Fishes from Matica R., although forming a special group, were included among other populations from the Neretva R. basin (with the exception of the Mostarsko blato population), which occupy the largest part of the plot. The plot of factor scores for males revealed a similar situation: specimens from Cetina, Krenica and the majority of those from Mostarsko blato formed separate groups, while those from the remaining populations were more alike.

### Results of Meristic Analysis

In contrast with the morphometric features, the number of fin rays did not show any difference between females and males from the same population. However, differences in meristic characters were noticed among different populations (Table 2). The number of branched fin rays was constant only in pelvic fins, where 5–7 (usually 6) rays were counted in all populations. On the other hand, the greatest variability was recorded in the dorsal fin rays number.

**Table 2:** Number of branched fin rays in specimens from investigated populations.

Population/locality (species)	Number of branched fin rays				
	dorsal	anal	caudal	pectoral	pelvic
Jadova (*C. jadovaensis*)	7^1^/_2_	6^1^/_2_ (7^1^/_2_)	(14) 15 (16)	10 (11)	6
Zrmanja (*C. bilineata*)	6^1^/_2_–7^1^/_2_	5^1^/_2_	14	8 (9)	6–7
Cetina (*C. dalmatina)*	6^1^/_2_–7^1^/_2_	5^1^/_2_	14 (15)	(8) 9 (10)	6 (7)
Metković (Neretva) (*C. narentana*)	7^1^/_2_	5^1^/_2_	14	8 (9)	6
Mislina (Neretva) (*C. narentana*)	7^1^/_2_ (8^1^/_2_)	5^1^/_2_	14	8–9	6
Norin (*C. narentana*)	(6^1^/_2_) 7^1^/_2_	5^1^/_2_	14	8–9	6
Modro oko (*C. narentana*)	(6^1^/_2_) 7^1^/_2_ (8^1^/_2_)	5^1^/_2_	14	8 (9)	6 (7)
Hutovo blato (*C. narentana*)	(6^1^/_2_) 7^1^/_2_	5^1^/_2_	14	(8) 9	6
Trebišnjica (*C. narentana*)	(6^1^/_2_) 7^1^/_2_	5^1^/_2_	14	(8) 9	(5) 6
Matica (*C. illyrica*)	6^1^/_2_	5^1^/_2_–6^1^/_2_	14	(8) 9	6
Prološko blato (*C. illyrica*)	6^1^/_2_	5^1^/_2_ (6^1^/_2_)	14	(8) 9 (10)	6
Krenica (*C. illyrica*)	(5^1^/_2_) 6^1^/_2_	5^1^/_2_	(13) 14	(8) 9	5–6
Mostarsko blato (*C. herzegoviniensis* Buj & Šanda, sp. nov.)	6^1^/_2_	5^1^/_2_ (6^1^/_2_)	14	9 (10)	5–6

### Features of External Morphology

Generally, external morphology of spined loaches from different rivers and lakes was quite similar. Moreover, some details of external morphology traditionally used to differentiate between species turned out to include a certain amount of intraspecific and intrapopulational variability (Table 3).

**Table 3:** The external morphology characters in investigated populations.

Population (species)	Basic color	Spots on the caudal fin base	Gambetta zones	Dorsal blotches	Position of the suborbital spine	Suborbital spine	Scale coverage
Jadova R. (*C. jadovaensis*)	yellow	One dark small and oblique spot in upper half of the caudal fin base; very rarely a second, oval and lighter, spot can be seen beneath the first one. Pigmentation in deeper layer is very pale and amorphous.	Great intrapop. diversity. Various blotches in surface layer in Z4 (rarely amorphous), sometimes merged together in a stripe. Narrow, paler stripe in a lower pigmentation laye	High degree of intrapopulational variability recorded in all populations varying from clearly separated and regularly shaped blotches located on lighter or darker surface, to completely merged ones.	Hidden under the skin surface.	Consist out of two arms, joint in more-less square angle. The longer arm is positioned bellow the eye and usually ends with two pointed branches. The shorter arm is parallel with the anterior edge of the eye and ends with 1–3 branches.	Small, but clearly visible, scales throughout the body surface.
Zrmanja R. (*C. bilineata)*	bright yellow	Two clear dark oblique spots, the upper one usually darker and more oblique. Exceptions (lower spot paler and vertical, or both spots oval) extremely rare. Deeper layer of pigmentation absent.	Intrapop. diversity noticed; squares and rectangles in Z4 (surface layer) are usually completely separated. Deeper pigmentation layer usually presented by a narrow stripe.		Hidden under the skin surface.		Miniature scales inserted into the skin. They can be noticed behind the operculum and in front of the caudal fin.
Cetina R. (*C. dalmatina*)	very pale yellow	Usually no spots in surface layer. Even when some kind of spots can be noticed on the caudal fin base, they are not darker from the spots in the Gambetta zones, most often are located in the middle part of the body, irregular in shape and belong to deeper layer.	Great intrapop. diversity. Blotches in surface layer in Z4 various in shape, separated or merged together. Deeper layer consists of a narrow line.		Hidden under the skin surface		Small and scarce scales visible on the whole body surface.
Neretva R. in Metković (*C. narentana*)	yellow	Two spots are present–the upper one is darker and oblique, the lower one paler and oval (sometimes slightly oblique or irregular in shape). Exceptions (lower or both spots small and hard to notice, or upper spot vertical or irregular) are rare. Pigmentation in deeper layer amorphous and pale, often absent.	Great intrapop. diversity. Surface pigmentation layer in Z4 consists of big rectangles or squares, often merged together. Deeper layer usually looks like a narrow, pale line, but sometimes is thicker and darker.		Largely covered by skin, but with apex sometimes peeking out.		Scales pronounced, especially on the ventral parts, somewhere even overlapping.
Neretva R., Mislina (*C. narentana*)	yellow						
Norin (*C. narentana*)	yellow						Small scales.
Modro oko (*C. narentana*)	yellow						Scales pronounced, especially on the ventral parts, somewhere even overlapping.
Trebišnjica R. (*C. narentana*)	yellow				Greatly uncovered.		Small scales.
Hutovo blato (*C. narentana*)	yellow				Largely covered by skin, but with apex sometimes peeking out.		Scales pronounced, especially on the ventral parts, somewhere even overlapping.
Matica R. (*C. illyrica*)	yellow	Usually one dark spot belonging to the surface pigmentation layer located in the upper half of the caudal fin base. The exceptions are more frequent. Usually the spot is oblique; rarely it has a comma shape, or is vertical or oval. Very rarely it is small or hard to notice and pale, while exceptionally it cannot be noticed at all. Deeper layer developed better than in other populations, presented by one or more paler spots, oval or irregular in shape.	Great intrapop. diversity. Blotches in the surface layer in Z4 rectangular, oval or irregular in shape; sometimes partly or completely merged together. Narrow, pale pigmentation line present in deeper layer, rarely thicker and/or darker.		Largely covered by skin, but with apex sometimes peeking out.		Miniature scales (visible under magnification).
Prološko blato (*C. illyrica*)	yellow				Greatly uncovered.		Small scales inserted into the skin.
Krenica (*C. illyrica*)	yellow				Hidden under the skin surface.		Miniature scales (visible under magnification).
Mostarsko blato (*C. herzegoviniensis* Buj & Šanda, sp. nov.)	yellow	No spots in the upper layer. Amorphous pigmentation in deeper layer developed.	Intrapop. diversity noticed. Squares in the surface layer in Z4 usually partially or completely merged, sometimes forming a dark stripe. Deeper pigmentation layer presented by dark line, sometimes darker than blotches in the surface layer.		Hidden under the skin surface.		Miniature scales (visible under magnification).

### Phylogeny of Dalmatian and Herzegovinian *Cobitis* Populations

Out of 122 tissue samples used for molecular analyses we have obtained satisfactory cyt *b* sequences from 120 samples, RAG1 sequences from 99 samples and S7 first intron from 80 samples. Thereafter, molecular phylogenetic analysis included 120 new cyt *b* sequences of length 1140 base pairs (bp), 198 nuclear RAG1 sequences (903 bp) and 160 S7 sequences (510 bp including indels). For cyt *b*, 59 different haplotypes were found among Dalmatian and Herzegovinian specimens, while for RAG1 and S7, 35 unique haplotypes were obtained for each gene. Of the samples analyzed, 68% were homozygous for RAG1, while only 36% were homozygous for the S7 first intron. The greatest number of individuals was phased with high probabilities ( = 1.00). Three individuals were phased with lower probabilities (0.51–0.83) in one or more heterozygous sites in RAG1. Inside the S7 data set, twelve individuals were phased with lower probabilities (0.51–0.84) for one or more heterozygous positions and others with high probabilities (>0.94). In both data sets, besides best haplotype guesses, all other haplotype possibilities were checked for ambiguous sites (with probabilities <0.94). Since ambiguous sites did not represent parsimony informative, nor diagnostic characters, they turned out not to be important in phylogenetic reconstructions so phylogenetic analyses were based on all alleles. The χ^2^ test for base homogeneity indicated that base frequency distributions were always homogenous among taxa for all three genetic markers. Table 4 reports the phylogenetic performance of each gene. Neutrality tests suggested almost no deviation from mutation–drift equilibrium for all genes analyzed; Fu & Li’s D* and F* statistics, as well as Tajima’s D were not statistically significant (p>0.05 for all investigated genes and species, with exceptions of Fu & Li’s F* and Tajima’s D for cyt *b* in *C. dalmatina*, Tajima’s D for S7 in *C. narentana* and Fu & Li’s D* and F* for S7 in the population from Mostarsko blato, where 0.02>p>0.05). The estimated minimum number of recombination events was much smaller than the number of mutations in nuclear genes (5 vs. 29 in RAG1, 4 vs. 52 in S7 first intron). Therefore, we believe that phylogenetic pattern was not violated by selective forces in any of the investigated genetic markers.

**Table 4:** Comparison of the phylogenetic performance of investigated genes.

gene	Total characters	Parsimony informative characters (in %)	Tv/Ts ratio	Length of the parsimony tree	Consistency index (CI)	Homoplasy index (HI)	Retention index (RI)
cyt *b*	1140	369 (32.36%)	1.66	1398	0.4514	0.5486	0.8789
RAG1	910	17 (1.87%)	1.44	104	0.8462	0.1538	0.8919
S7	511	32 (6.26%)	1.63	88	0.8182	0.1818	0.8678

**Figure 3:**
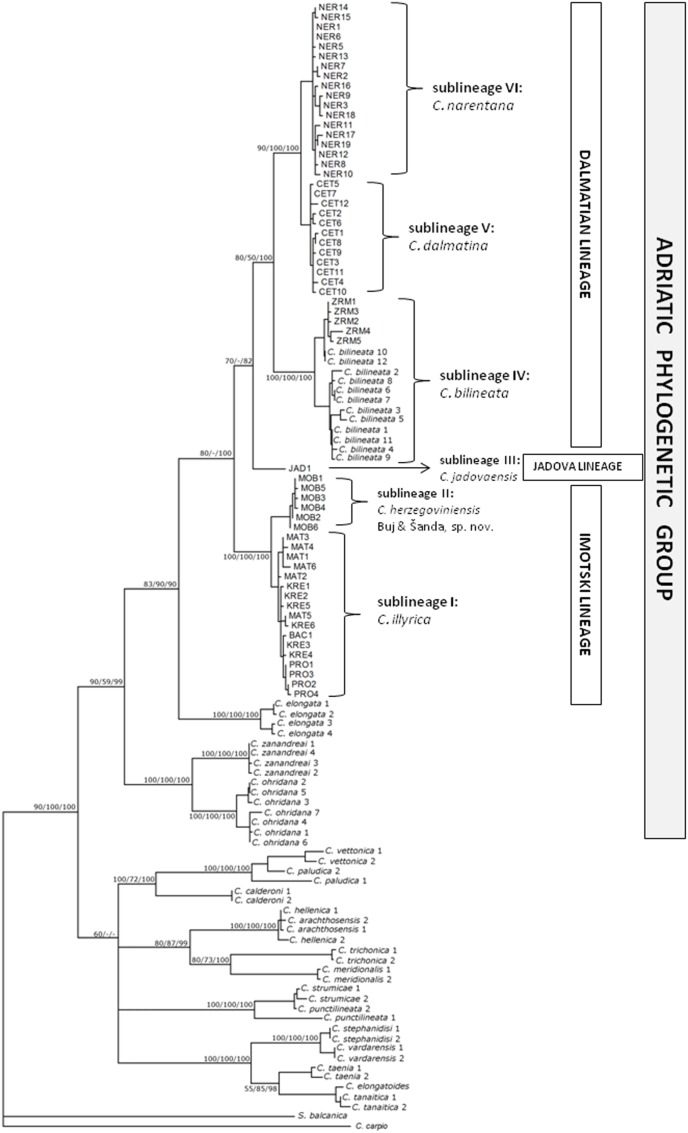
ML phylogram of cyt *b* haplotypes showing position of and phylogenetic relationships among Dalmatian and Herzegovinian spined loaches. Numbers at nodes represent ML BS, MP BS and BPP values. The species delimitation (each mtDNA sublineage = separate species) is based on the results of this investigation, and is not completely concordant with literature data.

All three methods of phylogenetic inference employed for cyt *b* sequences yielded trees with a similar overall topology (Figure 3). Samples from the investigated region, together with *C. bilineata* sequences retrieved from GenBank clustered as a monophyletic subgroup of the so-called Adriatic phylogenetic group (*sensu* [21]). Moreover, in accordance with previous investigations [21] [22], the Adriatic phylogenetic group also contained sequences of *C. elongata* Heckel & Kner, 1858, *C. ohridana* Karaman, 1928, and *C. zanandreai* Cavicchioli, 1965. Partition of this group and the position of nearly all internal branches was the same in trees obtained by different methods, with one exception–the position of the haplotype from Jadova R. In previous investigations subgroups were denoted as “bilineata”, “elongate” and “ohridana-zanandreai” clades [21] [22]. In this investigation we have analyzed relationships inside “bilineata” clade or subgroup.

**Figure 4:**
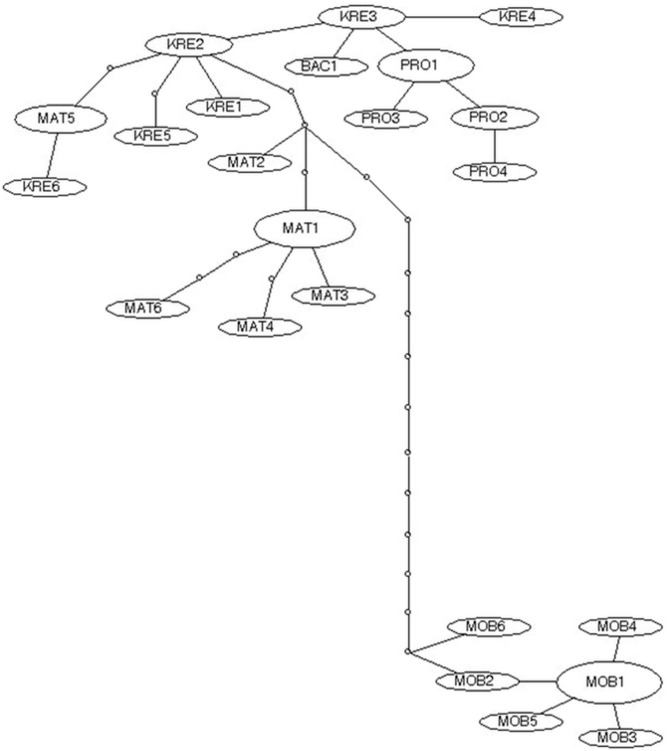
95% parsimony network of cyt *b* haplotypes of spined loaches from Matica R., Baćinska Lakes, Prološko blato, Krenica and Mostarsko blato. The size of ovals corresponds to haplotype frequency. Small circles are missing (unobserved) haplotypes.

The abovementioned subgroup of the Adriatic phylogenetic group, that comprises haplotypes from the investigated area, consists of three evolutionary independent lineages. One, hereafter named as “Imotski lineage”, comprised haplotypes from Matica, Baćinska lakes, Prološko blato, Krenica and Mostarsko blato, divided into two sublineages (a division clearly visible also on the SP network based on cyt *b* sequences from these localities; see Figure 4). The second lineage, named hereafter “Dalmatian lineage”, was divided into two branches. The first branch included all Zrmanja haplotypes, together with the Italian *C. bilineata* haplotypes, while the second branch comprised haplotypes from Cetina R. and Neretva R. basin (without Matica, Baćinska lakes, Krenica and Mostarsko blato), divided into two sublineages. The third lineage, named “Jadova lineage” was represented by the only haplotype found in the Jadova population. Based on ML and BAY analyses, this lineage is a sister group to the “Dalmatian lineage”, while in the MP phylogram it represents a sister lineage to the “Imotski lineage”.

**Figure 5:**
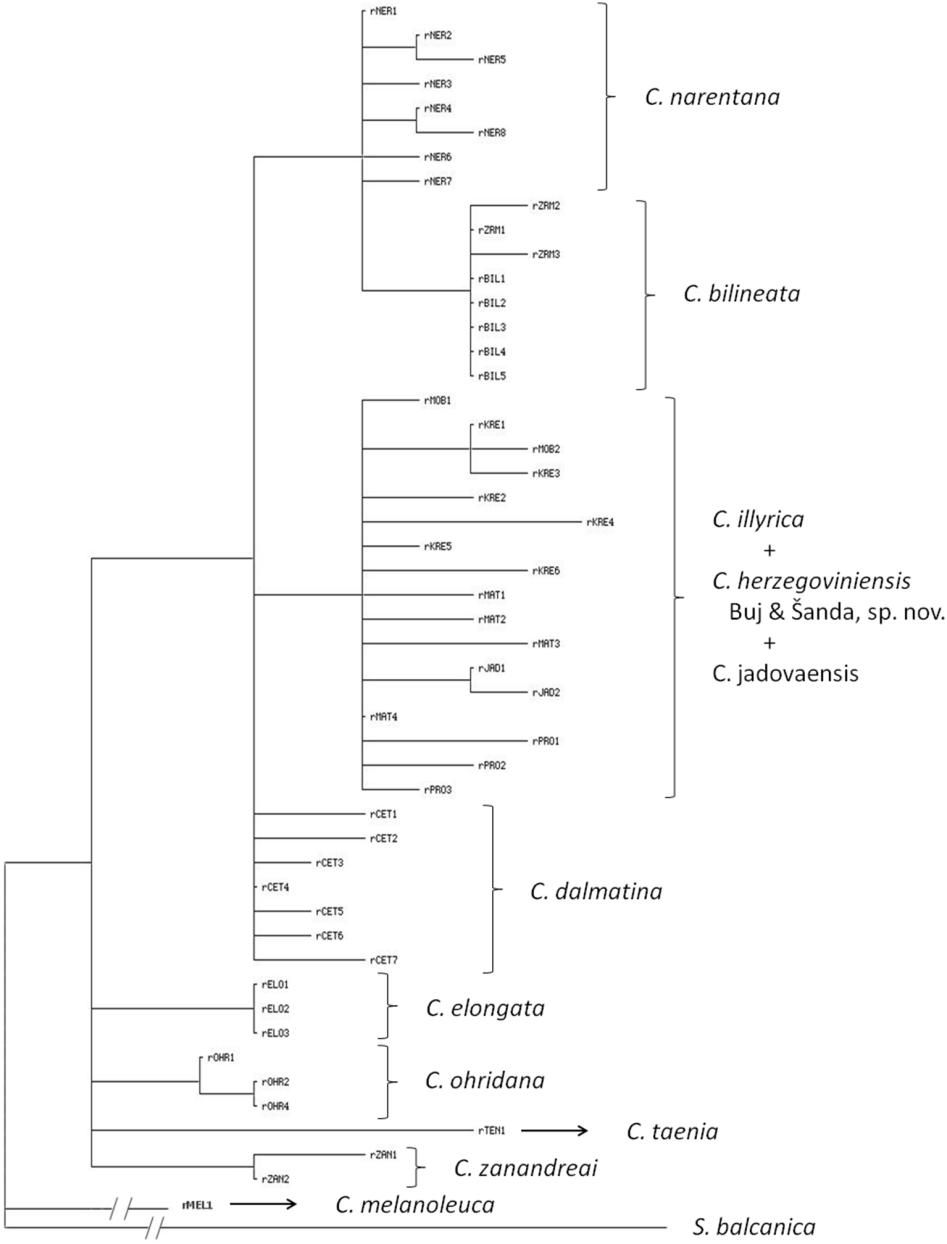
ML phylogram of RAG1 haplotypes. Numbers at nodes represent ML BS, MP BS and BPP values. Species delimitation is based on the results of this investigation, and is not completely concordant with literature data.

The phylogenetic relationships recovered by RAG1 haplotypes (Figure 5) partially differed from the cyt *b* phylogenies. Even though the taxon sampling was not the same (because of the fewer number of RAG1 sequences of non-Adriatic species available in GenBank), it is surprising that the only *C. taenia* L., 1758 RAG1 sequence available from GenBank clustered together with those of *C. ohridana* and *C. zanandreai*. Three subgroups of the Adriatic phylogenetic group were also recovered by RAG1, and all Dalmatian and Herzegovinian haplotypes were positioned inside one subgroup, as in the cyt *b* phylogenetic tree. Furthermore, “Imotski” and “Dalmatian lineage” were also recovered. However, the “Jadova lineage” was not recovered, yet Jadova haplotypes clustered inside “Imotski lineage”; haplotypes from the Cetina population did not cluster inside “Dalmatian lineage” and the internal structuring of the lineages was not resolved during analysis of the RAG1 gene, as it was in the cyt *b* phylogenies. The exception was *C. bilineata* (haplotypes from Zrmanja R. and those retrieved from GenBank), which was also monophyletic in RAG1 phylogenetic trees.

**Figure 6:**
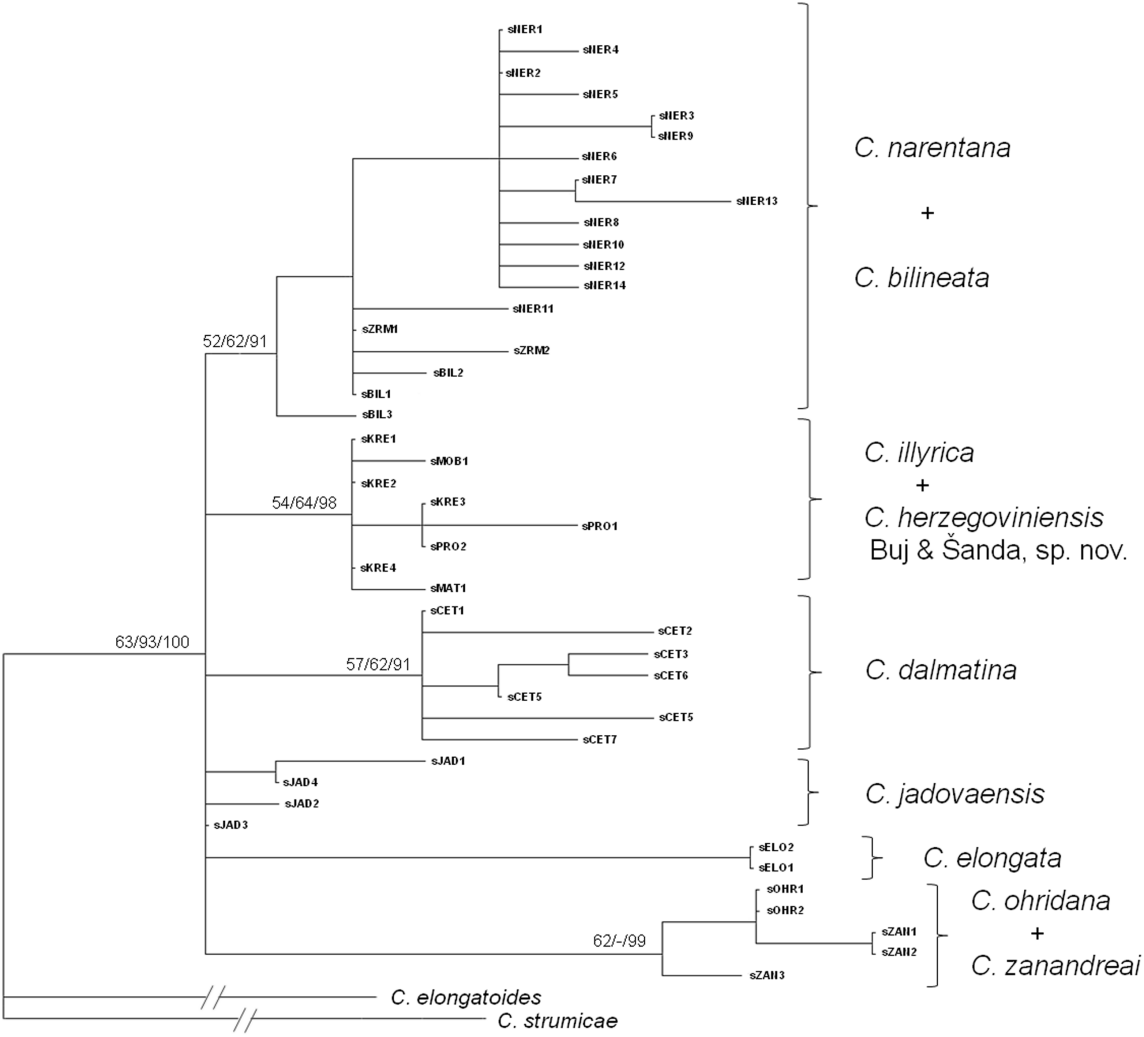
ML phylogram of S7 first intron haplotypes. Numbers at nodes represent ML BS, MP BS and BPP values. The species delimitation is based on the results of this investigation, and is not completely concordant with literature data.

In the phylogenetic reconstruction of the S7 first intron only two non-Adriatic species were included and they were used for rooting the trees. The differences between S7 phylogenetic trees (Figure 6) when compared to RAG1 phylogenies are in the position of the Jadova haplotypes (clustered here inside the “Dalmatian lineage”) and the monophyly of all haplotypes from Cetina R. The topology of the S7 phylogenetic trees was more similar to those obtained by phylogenetic reconstruction of cyt *b* haplotypes.

**Figure 7:**
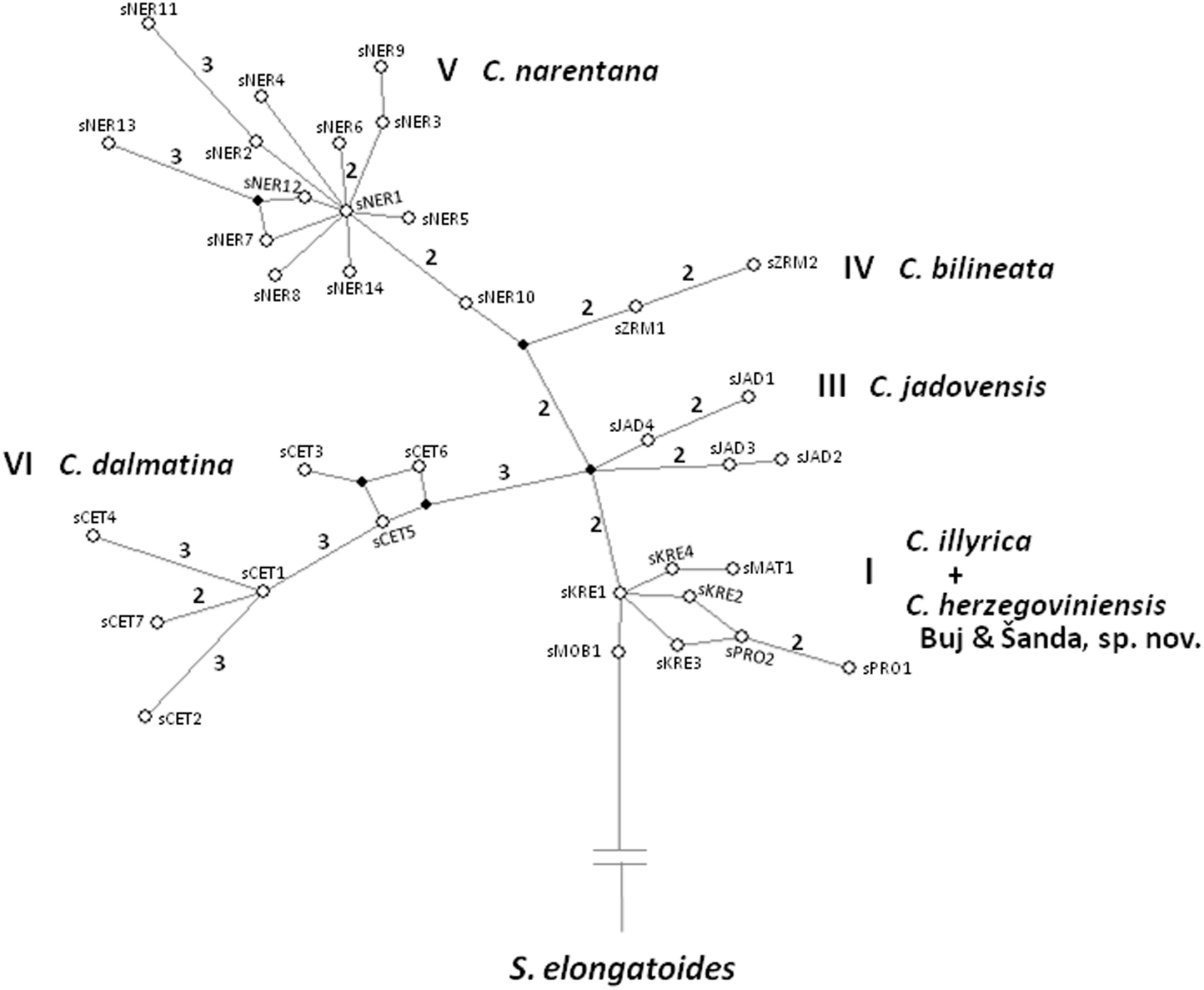
Median-joining network of S7 first intron haplotypes. Black circles represent median vectors. The number of mutational steps is displayed by the branches, when there were two or more mutations. Haplotype clusters are marked with roman numbers, corresponding to the numbers used to mark the mtDNA sublineages in Figure 3. The species designation is based on the results of this investigation.

**Figure 8:**
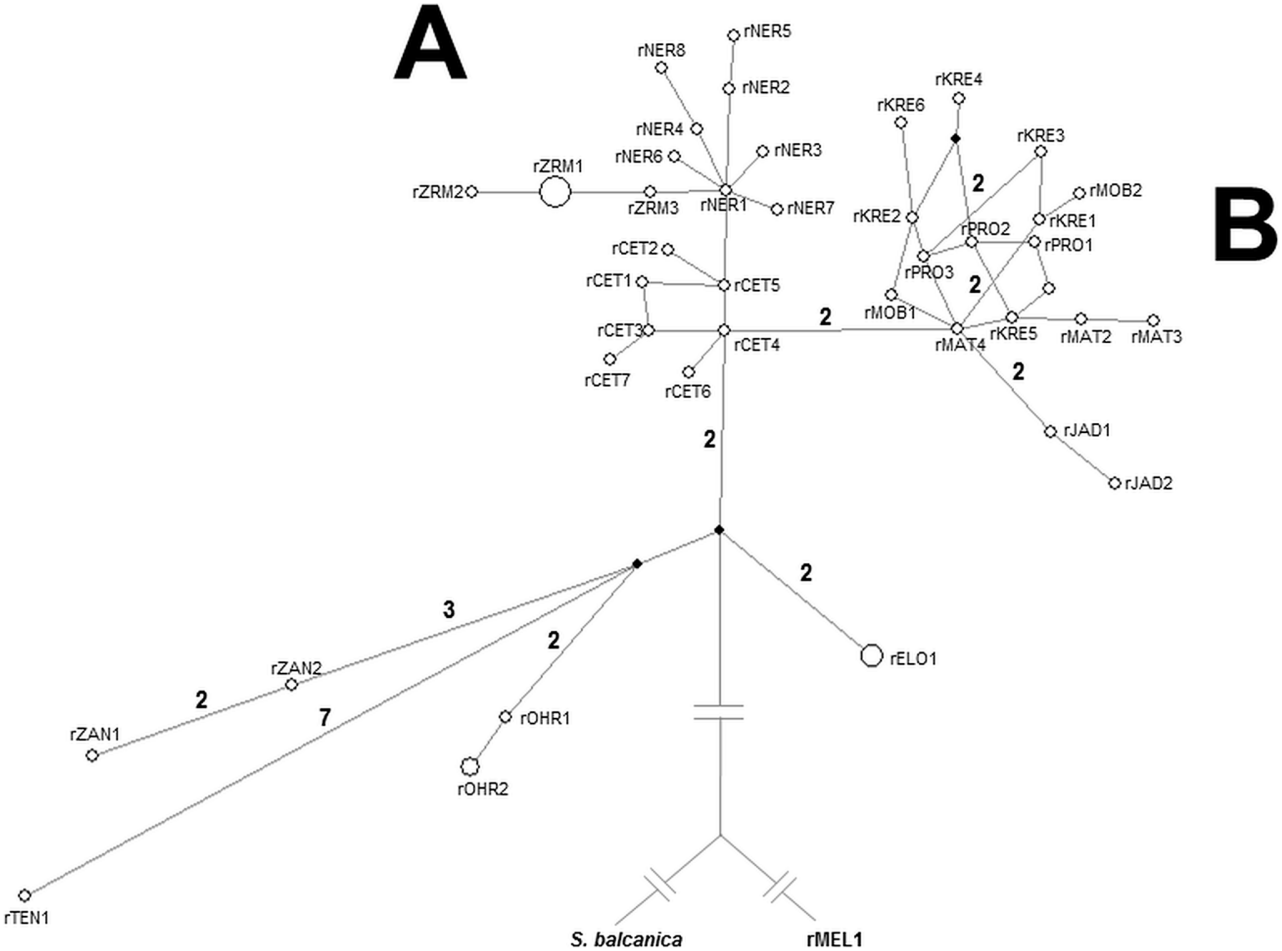
Median-joining network of RAG1 haplotypes. Black circles represent median vectors. The number of mutational steps is displayed by the branches, when there were two or more mutations. Haplotypes from the investigated area are grouped into two clusters: cluster A corresponds to the “Dalmatian mtDNA lineage” and cluster B to the “Imotski mtDNA lineage”.

A somewhat better resolution of phylogenetic relationships between investigated populations based on nuclear markers was obtained by MJ networks. In the phylogenetic network based on the S7 first intron (Figure 7), five clusters of haplotypes were separated, corresponding to the mtDNA sublineages. The difference is in the incorporation of haplotypes from the Mostarsko blato karstic field into the same cluster with the haplotypes from Matica, Baćinska lakes, Prološko blato and Krenica. In the RAG1 haplotype network (Figure 8), two clusters corresponding to “Imotski” and “Dalmatian” mtDNA lineages can be recognized, with haplotypes from Jadova R. forming an independent lineage inside “Imotski” cluster and haplotypes from Zrmanja R. forming a monophyletic lineage inside “Dalmatian” cluster.

It is important to mention that there was no sharing of mtDNA haplotypes between six sublineages recovered from the phylogenetic reconstruction of the cyt *b* gene. On the other hand, samples from Mostarsko Blato, besides unique nuclear haplotypes, possessed RAG1 and S7 haplotypes that were also found in individuals from Matica R., Krenica Lake and Prološko Blato Lake (haplotypes rKRE1 and sKRE1).

Interspecific p-distances based on cyt *b* gene ranged from 1.1 to 5.9%, while values of intraspecific p-distances were up to 0.9% (Table 5). Inter- and intra-specific p-distances of RAG1 were lower than those for cyt *b*, as expected (Table 5).

**Table 5:** Ranges and mean values (in brackets) of the p-distances in the cyt *b* (regular letters) and RAG1 genes (bold letters) among the investigated species, as well as the intraspecific p-distances.

	JAD	BIL	DAL	NAR	ILI	HER	Intraspecific cyt *b*	Intraspecific RAG1
JAD		**0.7–1.0 (0.8)**	**0.3–0.8 (0.6)**	**0.7–1.1 (0.8)**	**0.3–0.9 (0.6)**	**0.4–0.7 (0.6)**	0	0.1–0.2 (0.1)
BIL	4.0–4.6 (4.2)		**0.2–0.6 (0.4)**	**0.2–0.6 (0.4)**	**0.6–1.0 (0.8)**	**0.7–1.0 (0.8)**	0.1–0.6 (0.3)	0.1–0.2 (0.1)
DAL	3.3–3.7 (3.5)	3.4–4.2 (3.7)		**0.2–0.6 (0.4)**	**0.4–0.9 (0.6)**	**0.6–0.9 (0.7)**	0.1–0.9 (0.4)	0.1–0.2 (0.1)
NAR	3.4–3.9 (3.6)	3.6–4.6 (4.0)	1.1–1.8 (1.4)		**0.6–1.1 (0.8)**	**0.7–1.1 (0.8)**	0.1–0.7 (0.3)	0.1–0.3 (0.2)
ILI	3.4–3.9 (3.6)	4.9–5.9 (4.2)	4.4–5.1 (4.7)	4.4–4.9 (4.6)		**0.1–0.8 (0.5)**	0.1–0.9 (0.4)	0.1–0.9 (0.4)
HER	3.9–4.0 (3.9)	4.9–5.7 (5.2)	4.8–5.4 (5.0)	4.6–5.2 (4.9)	1.2–1.8 (1.5)		0.1–0.4 (0.2)	0.1–0.3 (0.2)

Abbreviations: JAD = *C. jadovaensis*; BIL = *C. bilineata*; DAL = *C. dalmatina*; NAR = *C. narentana*; ILI = *C. illyrica*; HER = *C. herzegoviniensis* Buj & Šanda, sp. nov.

### Bayesian Model-Testing

Results of model averaging, namely values of Bayes factors used to compare estimated marginal likelihoods of possible taxonomic groupings, are presented in the Table 6. Based on HME and SS methods for marginal likelihood estimations the model A is preferred over all other models, whereas based on PP method the model C is better than the remaining models. Nevertheless, log BFs among models A, C and D are lower than 3, a value that is considered as a strong support for one model over another [58].

**Table 6:** Bayes factors (BF) for different taxonomic groupings (models) investigated in *BEAST. Bold numbers mark the highest BF values and the most probable model, based on each employed method (HME-harmonic mean estimator, PS-path sampling, SS-stepping stone sampling).

HME	A	B	C	D	E	F
A		**4.57**	**1.15**	**0.38**	**7.31**	**7.66**
B	−4.57		−3.42	−4.19	2.74	3.09
C	−1.15	3.42		−0.77	6.16	6.51
D	−0.38	4.19	0.77		6.93	7.28
E	−7.31	−2.74	−6.16	−6.93		0.35
F	−7.66	−3.09	−6.51	−7.28	−0.35	
**PS**	**A**	**B**	**C**	**D**	**E**	**F**
A		3.2	−0.57	0.00	9.23	8.22
B	−3.2		−3.76	−3.19	6.03	5.02
C	**0.57**	**3.76**		**0.57**	**9.79**	**8.78**
D	−0.00	3.19	−0.57		9.22	8.21
E	−9.23	−6.03	−9.79	−9.22		−1.01
F	−8.22	−5.02	−8.78	−8.21	1.01	
**SS**	**A**	**B**	**C**	**D**	**E**	**F**
A		**4.94**	**1.9**	**2.2**	**9.6**	**8.34**
B	−4.94		−3.05	−2.74	4.69	3.4
C	−1.9	3.05		0.31	7.73	6.44
D	−−2.21	2.74	−0.31		7.43	6.13
E	−9.63	−4.69	−7.73	−7.43		−1.29
F	−8.34	−3.4	−6.44	-6.13	1.29	

Model A recognizes six different species; in model B *C. bilineata* is presented with two species (Zrmanja and Italy); in model C *C. illyrica* and *C. herzegoviniensis* Buj & Šanda, sp. nov. are considered as one species; model D is similar, but *C. dalmatina* and *C. narentana* are also considered as one species; model E relies on the RAG1 phylogeny; model F incorporates S7 first intron phylogeny.

### Molecular Diagnostics

Once the taxonomic status of Dalmatian and Herzegovinian populations was estimated from morphological and phylogenetic analysis, fixed differences and shared polymorphisms among species were revealed in all three molecular markers investigated (Table 7). Diagnostic sites in cyt *b* were found in all species (Table 8) enabling molecular identification of each species. RAG1 gene enabled molecular identification of *C. jadovaensis* (A at position 444) and *C. bilineata* (T at 852), while based on the S7 first intron *C. bilineata* (two diagnostic sites: 487, T; 502, A) and *C. dalmatina* (190, G) could be determined. In cyt *b* and RAG1 genes all mutations were substitutions. Insertions and deletions were found only in the S7 first intron. For the purposes of molecular diagnostics, the most important insertions and deletions are a single base insertion (T) at position 317 in *C. jadovaensis* and deletion of a segment comprising eleven nucleotides (positions 335–345) in *C. narentana*. Even though these mutations were found in most specimens of those species, they cannot be considered apomorphic characters because they were not present in all sequences. Moreover, we found individuals of both species that had one allele with and the other without mutation.

**Table 7:** The number of fixed differences (regular letters) and shared polymorphisms (bold letters) among the Adriatic *Cobitis* species in cyt *b*/RAG1/S7 first intron.

	JAD	BIL	DAL	NAR	ILI	HER
JAD		**0/0/0**	**1/0/0**	**0/0/1**	**0/0/1**	**0/0/0**
BIL	46/6/4		**0/1/0**	**1/0/0**	**1/0/0**	**0/0/0**
DAL	34/3/2	34/2/6		**0/0/1**	**2/0/0**	**0/0/0**
NAR	36/6/2	38/1/2	7/1/3		**4/0/0**	**0/0/0**
ILI	36/2/0	53/4/4	43/2/2	44/4/2		**0/3/0**
HER	46/2/2	56/4/6	49/2/4	48/4/4	10/0/0	

Abbreviations: JAD = *C. jadovaensis*; BIL = *C. bilineata*; DAL = *C. dalmatina*; NAR = *C. narentana*; ILI = *C. illyrica*; HER = *C. herzegoviniensis* Buj & Šanda, sp. nov.

**Table 8:** Diagnostic characters in the cyt *b* gene for the Adriatic *Cobitis* species.

species	position	base
*C. jadovaensis*	102	T (vs. C, A)
	444	C (vs. T)
	588	T (vs. C)
	642	T (vs. C)
	960	C (vs. T)
	982	T (vs. C)
	1047	C (vs. T)
*C. bilineata*	201	C (vs. T)
	213	C (vs. T)
	265	C (vs. T)
	276	T (vs. C)
	360	C (vs. T)
	477	T (vs. C)
	478	T (vs. C)
	501	T (vs. A, G)
	546	T (vs. C)
	600	T (vs. C)
	631	C (vs. T)
	675	G (vs. A)
	924	C (vs. T)
	987	T (vs. C)
	1023	A (vs. G)
*C. dalmatina*	447	T (vs. C)
*C. narentana*	52	T (vs. C)
	219	T (vs. G, A)
	267	C (vs. T)
	580	C (vs. A)
	897	A (vs. G)
*C. illyrica*	352	G (vs. A)
	1005	G (vs. A)
*C. herzegoviniensis* Buj & Šanda, sp. nov.	132	G (vs. A)
	318	C (vs. T, A)
	327	C (vs. T)
	660	C (vs. T)
	954	G, C (vs. A)

## Discussion

### Taxonomy and Distribution of Dalmatian and Herzegovinian Spined Loaches

Based on phylogenetic reconstruction of mtDNA haplotypes, observed morphological differences and genetic distances we conclude that each mtDNA sublineage (with the exception of *C. bilineata* sublineages, as discussed later) represents a separate *Cobitis* species. A large number of investigations conducted on different fish species have found that the mutation rate of the cyt *b* gene makes it suitable for taxonomic studies at the level of species. The present investigation confirms that the phylogenetic performance of the cyt *b* gene is more adequate for such research in comparison with both investigated nuclear genes, because it offers a larger number of parsimony informative sites and exhibits a lower consistency index (Table 4). This conclusion was also obtained by Perea et al. [59] based on study of species from the Leuciscinae subfamily. Phylogenetic reconstructions based on nuclear genes are partly concordant with mtDNA phylogenies, while recorded discrepancies are most probably the result of incomplete lineage sorting. Namely, several comparative investigations have indicated that monophyly, or exclusivity at the level of nuclear DNA, is not a necessary assumption for species that have diverged more recently, due to the slower divergence of nuclear genes in comparison to mitochondrial genes [60]–[62]. Comparing the phylogenetic performance of two nuclear genes, we can conclude that S7 first intron is more appropriate for phylogenetic investigations at species level (due to a faster evolution rate and a greater number of parsimony informative characters), while RAG1 is more suitable for higher taxonomic levels. The alternative hypothesis for differences in mtDNA and nuclear phylogenies, especially in the presence of the same nuclear haplotype in individuals belonging to different mtDNA lineages, would include hybridization events. Even though that hypothesis requires further attention, we found that scenario less likely. Namely, shared haplotypes were found in homozygote individuals of both species. Furthermore, we did not find a single individual with private haplotypes of different species. Thereafter, we believe that the observed pattern is more consistent with hypothesis that the divergence of species that share some nuclear haplotypes happened more recently so that each still contains a portion of ancestral alleles. Nevertheless, a possibility of hybridization cannot be rejected without further investigation and it is also possible that both phenomena (incomplete lineage sorting and hybridization) can be traced in the evolutionary history of the Adriatic spined loaches.

Even though we have calculated BF using three methods of marginal likelihood estimation, the model averaging could not be considered as decisive in our case due to the low BF values among the most probable models. Based on the results of this analysis, models E and F (groupings based on nuclear genes phylogenies) can be positively ruled out, which also speaks in favor to the conclusion that cyt *b* gene is more adequate for research of taxonomic relationships on the species level. On the other hand, positive decision regarding models A (six species), B (five species) and C (four species) cannot be made using this approach. Nevertheless two (HME and PS) of three employed methods recognized model A as the most likely one, which is concordant with our opinion based on all other results. When PS was used for marginal likelihood estimation, model C turned out as better than A with low BF (0.57), whereas models A and D were the same (BF = 0.00). In our investigation, stepping stone method for marginal likelihood estimation enabled the best resolution (highest BF factors) of the competing models. Nevertheless, even though this concrete case also corroborates usefulness of the recently described approach using BF in species delimitation; it also highlights its constraints (we could not obtain positive decision between three most likely scenarios, PS and SS method gave different results) and the importance of the systematic biologists’ critical opinion in taxonomic investigation, especially in cryptic species delimitation process.

Consequently it can be concluded that the region of Dalmatia and Herzegovina is in fact inhabited by six *Cobitis* species:

- *C. jadovaensis* distributed only in Jadova R.
- *C. bilineata* inhabiting Zrmanja R. in Croatia.
- *C. dalmatina* restricted to Cetina R.
- *C. narentana* inhabiting lower parts of Neretva R. with its tributaries and channels (Norin and Mislina) and Modro oko Lake in Croatia, as well as Trebišnjica R. and the Hutovo blato wetland in Bosnia and Herzegovina.
- *C. illyrica* distributed in Baćinska Lakes, Matica R., Prološko blato and Krenica.
- *C. herzegoviniensis* Buj & Šanda, sp. nov. from Mostarsko blato, a species described in this paper.

Our results reject the assumption that *C. narentana* inhabits Matica R. [27] and reveals that its distribution is considerably smaller than previously reported. We also found that *C. illyrica* is not restricted only to the Imotsko polje karstic field [15] but also inhabits waters of Polje Jezero karstic field in Croatia, and Bekijsko polje in Bosnia and Herzegovina.

*Cobitis jadovaensis* is clearly separated from the other species both genetically and morphologically. Besides the appearance of spots on the caudal fin base, this species differs from the others also in the number of branched rays in the anal, caudal and pectoral fins. An individual position was also supported by phylogenetic reconstructions based on cyt *b* and S7 first intron. Molecular diagnostic characters were found in cyt *b* and RAG1 genes. Furthermore, the intraspecific p-distance of this species is much less than the p-distance between *C. jadovaensis* and the other species.

In our study *Cobitis bilineata* was genetically (in all investigated genes) and morphologically (especially in the appearance of caudal fin base spots, but also in overall coloration) clearly separated from the remaining species. The p-distance between this and the remaining Dalmatian species is quite high, though phylogenetic analysis revealed it is more closely related to *C. narentana* and *C. dalmatina*. However, extraordinary deep splitting and also high intraspecific p-distance was recorded for *C. bilineata*. Namely, the p-distance between two sublineages comprised by this species (Figure 3) is 0.9–1.5%, a level higher than intraspecific p-distances of all the remaining investigated species. In fact, p-distances recorded inside each *C. bilineata* sublineage resemble intraspecific p-distances for Adriatic spined loaches. One of the sublineages comprises all haplotypes from Zrmanja R. together with two haplotypes from the Italian Reno R, while the second sublineage incorporates all the remaining Italian haplotypes, including two haplotypes from Reno R. Therefore, these two sublineages cannot be considered as two different species. Their independent evolutionary development was probably interrupted by secondary contact that disabled speciation.

The taxonomic status of spined loaches from Cetina and Neretva rivers has until now been uncertain due to the small genetic distance between them [21], [22]. However, the present investigation included a sufficient number of specimens to be assured of their independent evolutionary course and the distinct position of each was corroborated also by the nuclear DNA phylogenies. Cyt *b* provides diagnostic characters for both species, and *C. dalmatina* can also be determined based on the presence of guanine in position 190 of the S7 first intron. The p-distances between *C. dalmatina* and *C. narentana*, even though less than that between most of the species investigated, are still larger than the intraspecific p-distances. Also, fixed differences between these two species are present in all investigated genes, even though in lower numbers than among other species. *Cobitis dalmatina* and *C. narentana* can be distinguished based on the appearance of spots on the caudal fin base. Furthermore, morphometric analysis pointed to the separation of Cetina spined loaches from the others.

A similar, quite small, genetic distance was recorded between *C. illyrica* and *C. herzegoviniensis* Buj & Šanda, sp. nov. Nevertheless, the interspecific difference in cyt *b* is again larger than the intraspecific p-distances. There are also morphological differences that separate these two species, especially the appearance of spots on the caudal fin base. The morphometric analysis implied the separation of the Mostarsko blato population from all the remaining ones as well. Furthermore, the assumption that the Mostarsko blato population actually represents a separate species was confirmed by the phylogenetic network obtained by statistical parsimony analysis of all cyt *b* sequences consisting under the “Imotski phylogenetic lineage”. Haplotypes from Matica, Baćinska lakes, Prološko blato and Krenica are separated by up to three evolutionary steps (mutations), while spined loaches from Mostarsko blato are thirteen or more steps apart (Figure 4). Although nuclear genes do not enable discrimination between these two species, cyt *b* provides diagnostic sites for them. Moreover, there are ten sites in cyt *b* that represent fixed differences between them. In order to explain the discrepancies observed between phylogenies based on different genes, a further investigation of the evolutionary history of these species is required. Nevertheless, the S7 first intron phylogeny offers a preliminary hypothesis: it seems that the sMOB1 haplotype, unique to the Mostarsko blato population, is the basal haplotype for the “Imotski lineage” (Figure 7). The sKRE1 haplotype diverged from sMOB1 and is the only haplotype present in both species. Based on the S7 first intron MJ network (Figure 7), all *C. illyrica* haplotypes diverged from sKRE1, implying a possible colonization of the *C. illyrica* distribution range from the Mostarsko blato karstic field with individuals carrying sKRE1. Due to the slower mutation rate of nuclear genes in comparison with cyt *b*, in each species unique cyt *b* haplotypes evolved during isolation, while both species still contain some same nuclear haplotypes, identified as an example of incomplete lineage sorting.

Among six Dalmatian and Herzegovinian *Cobitis* species, only *C. bilineata* has a wide distribution; all the others are endemics with restricted distribution ranges. Such a large number of endemic species distributed in a small area, the presence of distinct species in neighboring localities and differences in the level of genetic diversity among species from the same area imply a complex evolutionary history of *Cobitis* in Dalmatia and Herzegovina, requiring further, especially population genetic, analysis.

### Diagnostic Characters for Adriatic *Cobitis* Species

#### Morphometric characters

Until the present paper, a detailed comparison of morphometric characters of spined loaches from Dalmatia and Herzegovina had not been conducted. To differentiate between species, several authors used the ratio between the depth and the length of the caudal peduncle (h/lpc), the ratio between pectoral fin length and the distance between the base of the pectoral and pelvic fins (lp/(aV-aP)) [9], [15], head length in relation to its width (c/hco) and length of anal fin base in relation to standard length (lA/SL) [11], as well as the ratio of body height and standard length (H/SL), but for species outside of the Adriatic basin [9]. The present investigation found that none of these characters have a diagnostic value due to their large intraspecific ranges and overlap between different species (Table S1). In addition to small and non-representative sample sizes, difficulties for previous investigations lay in analyses based upon samples comprising both females and males where the values being compared largely depend on the sex ratio in the sample. However, the results of this investigation found no morphometric character to have a diagnostic value alone and therefore to be used to discriminate any species. On the other hand, detailed comparison of all standardized morphometric features pointed to the same taxonomic conclusions as the molecular phylogenetic analyses. Besides differences in body shape between some populations (in particular the population from Cetina R., and also those from Prološko blato, Krenica and Mostarsko blato) and the remaining populations, PCA also led to the conclusion that the group comprising *C. narentana* populations is, based on morphometric characters, the most diverse group. Furthermore, the intrapopulational diversity of the Neretvanian loaches is similar to their interpopulational diversity, while the interpopulational diversity of *C. illyrica* is much higher than its intrapopulational diversity.

#### Meristic characters

Even though meristic characters have only rarely been used for *Cobitis* species determination we noticed phylogenetic signals in the number of branched fin rays (Table 2). Dorsal fin rays number can enable discrimination of *C. narentana* and *C. illyrica*. Furthermore, based on meristic characters, *C. jadovaensis* can be separated clearly from the remaining species.

#### External morphology

Among all investigated morphological features, only the appearance of spots on the caudal fin base turned out to be of diagnostic value. Although the appearance of Gambetta zones have been used to determine several *Cobitis* species [9], [11], [15], based on our results, the intrapopulational variability and similarities in the surface pigmentation layer among different species prevent the use of this character as a diagnostic marker. On the other hand, the deeper layer is presented with a narrow line in all species. Variability of the deeper layer was also recorded inside several populations. The same situation was noticed for dorsal blotches, a feature that also differs among specimens from the same population, with similar patterns recorded in the majority of populations.

**Figure 9:**
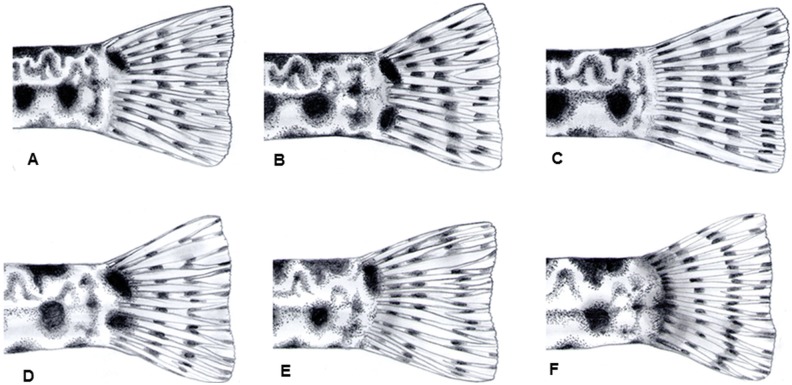
Pictures of the caudal parts of specimens from investigated *Cobitis* species. Legend: A = *C. jadovaensis*; B = *C. bilineata*; C = *C. dalmatina*; D = *C. narentana*; E = *C. illyrica*; F = *C. herzegoviniensis* Buj & Šanda, sp. nov. Characteristic spots on the base of the caudal fin can be seen.

The appearance of spots on the caudal fin base is the character most widely used for *Cobitis* species determination. Karaman [26] mentions this feature as a diagnostic character for *C. dalmatina* and *C. narentana*. Since then many authors have used this feature for determination of several spined loaches [9], [11], [12] [14], [15]. Although results obtained in the present investigation are not completely in accordance with the literature, with some intraspecific and intrapopulational diversity recorded, this feature turned out to be the best phenotypic diagnostic character (Table 3) enabling differentiation of all investigated species (Figure 9). The characteristic spots, that enable diagnosis of several species, are located in the surface pigmentation layer. Pigmentation in deeper layer is more variable, most often amorphous and pale. It is present in the majority of species, absent in *C. bilineata* and some specimens of *C. narentana*.

The position of the suborbital spine, namely its exposure vs. coverage by skin, differs among populations, even those of the same species. However, a constant pattern was noticed within each population, indicating this character’s connection with ecological conditions at the locality, as concluded for *Sabanejewia balcanica* (Karaman, 1922) [63]. On the other hand, we were able to reject the accepted view that all *Cobitis* species have a suborbital spine positioned on the skin surface [9], so that feature cannot be used for genus determination.

#### Molecular diagnostic characters

For the purpose of molecular diagnostics and barcoding, we propose utilization of the gene for cytochrome *b* that provides diagnostic characters for all Adriatic *Cobitis* species (summarized in Table 8).

### Contribution to Fish Taxonomy

In contributing to the taxonomy and determination of *Cobitis* species, based on the results obtained here, we bring a description of a new species from Mostarsko blato karstic field and a diagnosis for other species from the investigated area, as well as a key for their determination.

**Figure 10:**
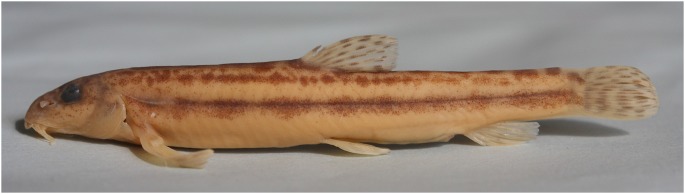
*Cobitis herzegoviniensis* Buj & Šanda, sp. nov., a newly described species from Mostarsko blato; holotype.

**Figure 11:**
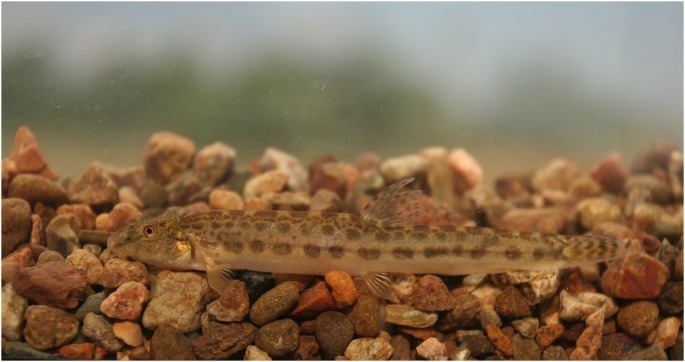
Photograph of a live specimen of *C. herzegoviniensis* Buj & Šanda, sp. nov.

***Cobitis herzegoviniensis*** Buj & Šanda, **sp. nov.** urn:lsid:zoobank.org:act: 24564E1D-1D17–4D9A–AD41-B9D030D06CB3 (Figures 10 and 11).

#### Holotype

NMP P6V 80904; ♂; 55.65 mm SL, the Lištica River in the Mostarsko blato karstic field, Bosnia and Herzegovina, N43°22′41.3″ E17°19′98.3″ elevation 227 m (leg. R. Šanda, A. Perdices, S. Perea, I. Bogut; 23.07.2004).

#### Paratypes

**Figure 12:**
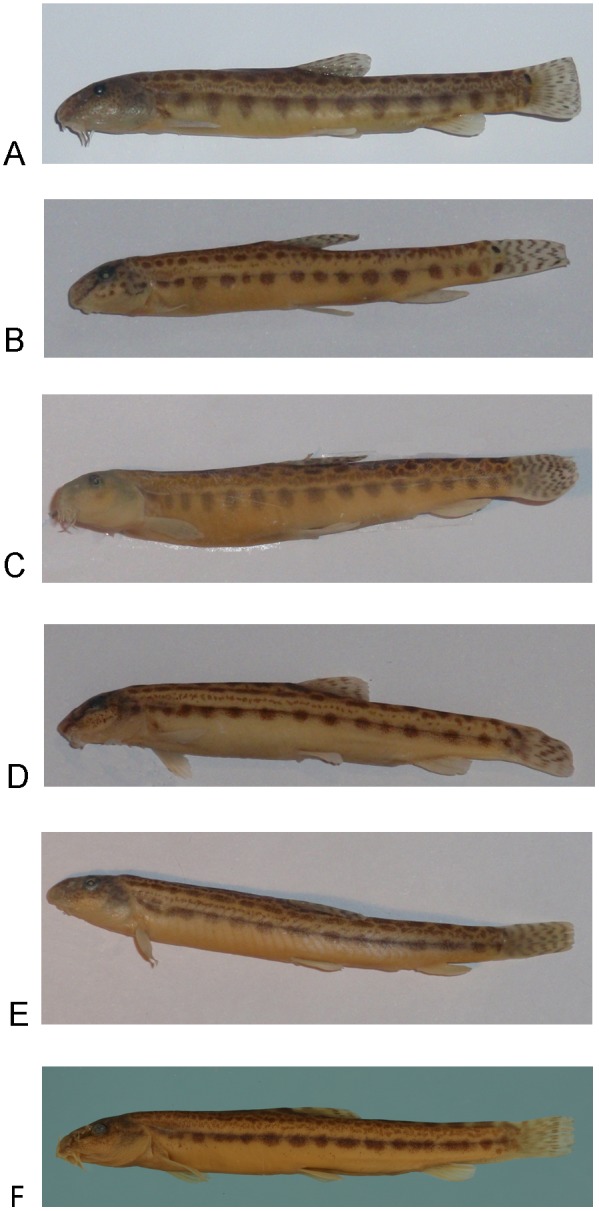
Photographs of representative individuals for each species. Legend: A = *C. jadovaensis*; B = *C. bilineata*; C = *C. dalmatina*; D = *C. narentana*; E = *C. illyrica*; F = *C. herzegoviniensis*, paratype PMR VP2950.

NMP P6V 80903, 80905, PMR VP 2950–52; data as for holotype; NMP P6V 81173, 75–77, 79; artificial channel in Mostarsko blato karstic field, Bosnia and Herzegovina, N43°19.360′ E17°43.099′ elevation 222 m (leg. R. Šanda, J. Kohout, A. Šedivá, I. Bogut, 13.07.2006); 5♂ and 5♀; 44.2–63.96 mm SL (Figure 12F).

#### Type locality

Lištica River in Mostarsko blato, Bosnia and Herzegovina.

ADDITIONAL MATERIAL: NMP P6V 81171, 72, 74, 78, 81180–90; 12♂ and 3♀; 51.47–76 mm SL; the same data as for paratypes NMP P6V 81173, 75–77, 79.

#### Etymology

The species name represents the name of the province where Mostarsko blato is located; an adjective.

#### Diagnosis

*Cobitis herzegoviniensis* Buj & Šanda, sp. nov. is distinguished from other species of the genus *Cobitis* by the following combination of characters: a single Canestrini scale on the pectoral fins of males, triangular in shape with roundish outer border; usually 5^1^/_2_ branched anal fin rays, 6^1^/_2_ branched dorsal fin rays, 14 branched caudal fin rays; no characteristic spots on the caudal fin base, but pale deeper pigmentation layer, irregular in shape, can sometimes be noticed; scales on the body very small, visible only under magnification.

#### Description

D II(III)+6^1^/_2_, A II–III+5^1^/_2_(6^1^/_2_), P 9(10), V I+5–6, C I+14+I. Body small, larger in females (TL 60–76 mm in females, 51–66 mm in males; SL 52–67 mm in females, 44–56 mm in males), very elongated. Body depth uniform throughout the whole body length; maximum body depth (in front of dorsal fin origin) 11.4–16.3% of SL. Caudal peduncle depth 47.2–71.5% of its length in females; 40.9–69% in males. Head very elongated, comprising 19.6–23% of SL. Very small (visible only under magnification), separated scales distributed throughout the body surface. Suborbital spine located below eyes and completely covered by a skin fold. Three pairs of barbels located around the mouth, variable in size. Pelvic fins shorter than the distance between pelvic fin base and anal aperture. Straight margins of dorsal and anal fins.

Males different from females in having longer pectoral fins (in males they occupy 65–78% of distance between pectoral and pelvic fins origins, while in females they occupy 28–45%), longer pelvic fins (10.8–15.6% of SL in males vs. 9.8–10.8% of SL in females), longer caudal fin (14.1–16.7% of SL in males vs. 12.5–15.4% of SL in females), longer head (19.6–23.0% of SL in males vs. 19.9–21.8% of SL in females) and the presence of a single Canestrini scale on a dorsal surface of pectoral fins. All the remaining body dimensions are larger in females than in males.

#### Coloration (preserved specimens)

Varying from distinctly yellow with dark black spots and blotches to somewhat paler overall coloration. The ventral parts yellow without pigmentation, or with few spots on fins. Small spots located also on the head, together with a stripe that usually ends in front of the eye, and rarely crosses the eye. Spots are often larger on the operculum and on the dorsal part of the head. Spots on the caudal fin base are not clearly visible and usually cannot be distinguished from the remaining body spots; even in specimens where spots (one or two) are noticed, they are small and hardly visible. On the body sides four Gambetta zones (Z1–Z4) can be recognized. Z1 is most often narrow and not pronounced, and is composed of very small, dense spots. In some specimens those spots are miniature and pale, even to the extent that they are hardly visible. Z2 consists of rectangular blotches that can be separated, but densely spaced, or merged into the single line. Z3 most often contains small pale spots. Often there are no spots in the posterior part of the Z3 and rarely Z3 is composed of only a few spots behind the head. Spots in Z3 are often located in a single line, rarely in several lines. In the majority of specimens Z4 is filled with square or rectangle blotches (presenting the surface pigmentation layer) on a darker line (located in the deeper pigmentation layer) that is usually more pronounced than blotches, while in posterior parts the blotches are interconnected into a single line. Sometimes, blotches are very pale and only a dark line is clearly visible. Dorsally, a single row of blotches is located, often irregular in shape and only sometimes squared. Dorsal blotches are sometimes separated, but located close to one another, and sometimes joined. Sexual dimorphism was not noticed in the coloration.

#### Comparative remarks

*Cobitis herzegoviniensis* Buj & Šanda, sp. nov. can be distinguished from *C. illyrica* by the appearance of spots on the caudal fin base (no pronounced spots in *C. herzegoviniensis* Buj & Šanda, sp. nov. vs. one spot in the surface pigmentation layer of the caudal fin base in *C. illyrica* (exceptionally those spots are hardly visible). *Cobitis herzegoviniensis* Buj & Šanda, sp. nov. differs from *C. narentana* in the number of branched rays in the dorsal fin (6^1^/_2_ in *C. herzegoviniensis* Buj & Šanda, sp. nov. vs. usually 7^1^/_2_ in *C. narentana*), the appearance of spots on the caudal fin base (no pronounced spots in *C. herzegoviniensis* Buj & Šanda, sp. nov. vs. two clearly visible spots in *C. narentana*). *Cobitis herzegoviniensis* Buj & Šanda, sp. nov. is different from *C. dalmatina* in the number of branched rays in the pelvic (5–6 in *C. herzegoviniensis* Buj & Šanda, sp. nov. vs. 6 (7) in *C. dalmatina*) and dorsal fin (6^1^/_2_ in *C. herzegoviniensis* Buj & Šanda, sp. nov. vs. 6^1^/_2_–7^1^/_2_ in *C. dalmatina*). *Cobitis herzegoviniensis* Buj & Šanda, sp. nov. can also be distinguished from *C. dalmatina* by the appearance of scales (miniature, visible only under magnification in *C. herzegoviniensis* Buj & Šanda, sp. nov. vs. small, but clearly visible in *C. dalmatina*). *Cobitis herzegoviniensis* Buj & Šanda, sp. nov. differs from *C. bilineata* in the appearance of spots on the caudal fin base (no pronounced spots in *C. herzegoviniensis* Buj & Šanda, sp. nov. vs. two clearly visible spots in *C. bilineata*). Differences between *C. herzegoviniensis* Buj & Šanda, sp. nov. and *C. jadovaensis* concern the appearance of spots on the caudal fin base (no pronounced spots in *C. herzegoviniensis* Buj & Šanda, sp. nov. vs. one spot in *C. jadovaensis*) and the number of branched rays in the dorsal (6^1^/_2_ vs. 7^1^/_2_), anal (usually 5^1^/_2_ vs. usually 6^1^/_2_), caudal (14 vs. usually 15) and pectoral fins (usually 9 vs. usually 10).

#### Distribution

*C. herzegoviniensis* Buj & Šanda, sp. nov. is endemic to Mostarsko blato karstic field in Bosnia and Herzegovina.

***cobitis jadovaensis*** Mustafić & Mrakovčić, 2004 (Figure 12A).

#### Diagnosis

*Cobitis jadovaensis* is distinguished from other species of the genus by the following combination of characters: a single triangular with roundish outer border Canestrini scale on the pectoral fins of males; usually 6^1^/_2_ branched anal fin rays, 7^1^/_2_ branched dorsal fin rays, usually 15 branched caudal fin rays, usually 10 branched pectoral fin rays; one small dark oblique spot located in the upper half of the caudal fin base, pigmentation in deeper layer not pronounced and irregular in shape; whole body covered with small, yet clearly visible, scales.

#### Comparative remarks

*Cobitis jadovaensis* differs from *C. bilineata*, *C. dalmatina*, *C. narentana*, *C. illyrica* and *C. herzegoviniensis* Buj & Šanda, sp. nov. by having usually 6^1^/_2_ (sometimes 7^1^/_2_) branched anal fin rays (vs. usually 5^1^/_2_); usually 15 branched caudal fin rays (vs. usually 14); usually 10 (vs. usually 8 or 9) branched pectoral fin rays. *Cobitis jadovaensis* can be distinguished from *C. bilineata*, *C. dalmatina*, *C. narentana* and *C. herzegoviniensis* Buj & Šanda, sp. nov. in the appearance of spots on the caudal fin base (usually one spot in *C. jadovaensis* vs. two spots or no pronounced spots).

#### Distribution

*C. jadovaensis* is endemic to Jadova River in Croatia.

***cobitis bilineata*** Canestrini, 1865 (Figure 12B).

#### Diagnosis

*Cobitis bilineata* is distinguished from the other species of the genus by the following combination of characters: a single triangular with roundish outer border Canestrini scale on the pectoral fins of males; 5^1^/_2_ branched anal fin rays, 14 branched caudal fin rays, usually 8 branched pectoral fin rays; two clear dark oblique spots on the caudal fin base, the upper one usually darker and more oblique; miniature scales inserted into the skin.

#### Comparative remarks

*Cobitis bilineata* is different from *C. jadovaensis*, *C. dalmatina*, *C. illyrica* and *C. herzegoviniensis* Buj & Šanda, sp. nov. in the appearance of the spots on the caudal fin base (two spots in *C. bilineata* vs. usually one or no pronounced spots). *Cobitis bilineata* can be distinguished from *C. narentana* by the appearance of the lower spot on the caudal fin base (usually oblique in *C. bilineata* vs. usually oval in *C. narentana*); overall coloration (bright yellow with clear dark spots in Gambetta zones in *C. bilineata* vs. paler body color and paler spots in *C. narentana*); and the appearance of spots in Z4 (usually clearly separated, big squares and rectangles in the surface pigmentation layer of the Z4 in *C. bilineata* vs. often partly or completely merged squares and rectangles, sometimes oval in shape in *C. narentana*).

#### Distribution in croatia

Zrmanja R. The distribution range of *C. bilineata* outside of Croatia was not investigated.

***cobitis dalmatina*** Karaman, 1928 (Figure 12C).

#### Diagnosis

*Cobitis dalmatina* is distinguished from the other species of the genus by the following combination of characters: a single triangular with roundish outer border Canestrini scale on the pectoral fins of males; usually 5^1^/_2_ branched anal fin rays, usually 14 branched caudal fin rays, usually 9 branched pectoral fin rays; no pronounced spots on the caudal fin base (no spots in the surface layer, but deeper pigmentation layer usually present); small scales on the whole body surface, but scarce.

#### Comparative remarks

*Cobitis dalmatina* can be distinguished from *C. jadovaensis*, *C. bilineata*, *C. narentana* and *C. illyrica* by the appearance of spots on the caudal fin base (no pronounced spots in *C. dalmatina* vs. 1 or 2 spots). *Cobitis dalmatina* differs from *C. herzegoviniensis* Buj & Šanda, sp. nov. in having 6–7 branched pelvic fin rays (vs. 5–6) and in having small, but clearly visible, scales on the body surface (vs. miniature scales visible only under magnification).

#### Distribution

*Cobitis dalmatina* is endemic to Cetina R. in Croatia.

***cobitis narentana*** Karaman, 1828 (Figure 12D).

#### Diagnosis

*Cobitis narentana* is different from the remaining species of the genus by the following combination of characters: a single Canestrini scale on the pectoral fins of males, triangular in shape with roundish outer border; 5^1^/_2_ branched anal fin rays, usually 7^1^/_2_ branched dorsal fin rays, 14 branched caudal fin rays, 8–9 branched pectoral fin rays; two spots on the caudal fin base–the upper one darker and oblique, the lower spot paler and usually oval; scales small or pronounced, but always visible.

#### Comparative remarks

*Cobitis narentana* differs from *C. jadovaensis*, *C. dalmatina*, *C. illyrica* and *C. herzegoviniensis* Buj & Šanda, sp. nov. in having usually 2 spots on the caudal fin base (vs. one spot or no clearly visible spots). It can be distinguished from *C. illyrica* and *C. herzegoviniensis* Buj & Šanda, sp. nov. by the number of branched dorsal fin rays (usually 7^1^/_2_ in *C. narentana* vs. usually 6^1^/_2_). *Cobitis narentana* is different from *C. bilineata* in the appearance of the lower spot on the caudal fin base (usually oval in *C. narentana* vs. oblique in *C. bilineata*); overall coloration (paler body color and paler spots in *C. narentana* vs. bright yellow body color with clear dark spots in Gambetta zones in *C. bilineata*); and the appearance of spots in Z4 (often partly or completely merged squares and rectangles, sometimes oval in shape in the surface pigmentation layer of the Z4 in *C. narentana* vs. usually clearly separated, big squares and rectangles in *C. bilineata*).

#### Distribution

*C. narentana* is distributed in the lower parts of Neretva R. with its tributaries and channels and Modro oko Lake in Croatia, as well as in Trebišnjica R. and Hutovo blato wetland in Bosnia and Hezegovina.

***cobitis illyrica*** Freyhof & Stelbrink, 2007 (Figure 12E).

#### Diagnosis

*Cobitis illyrica* can be distinguished from the other species of the genus by the following combination of characters: a single triangular with roundish outer border Canestrini scale on the pectoral fins of males; usually 5^1^/_2_ branched anal fin rays, usually 6^1^/_2_ branched dorsal fin rays, usually 14 branched caudal fin rays, usually 9 branched pectoral fin rays; usually one oblique spot in the upper half of the caudal fin base; small or miniature scales inserted into the skin.

#### Comparative remarks

*Cobitis illyrica* differs from *C. bilineata*, *C. dalmatina*, *C. narentana* and *C. herzegoviniensis* Buj & Šanda, sp. nov. in having usually one spot on the caudal fin base (vs. two spots or no spots). It is different from *C. narentana* in the number of branched dorsal fin rays (usually 6^1^/_2_ vs. usually 7^1^/_2_). *Cobitis illyrica* is different from *C. jadovaensis* in the number of branched dorsal fin rays (5^1^/_2_–6^1^/_2_ in *C. illyrica* vs. 7^1^/_2_ in *C. jadovaensis*); the number of branched caudal fin rays (usually 14 in *C. illyrica* vs. usually 15 in *C. jadovaensis*); the number of branched pectoral fin rays (usually 9 in *C. illyrica* vs. usually 10 in *C. jadovaensis*).

#### Distribution

*Cobitis illyrica* inhabits Matica R., Prološko blato and Baćinska Lakes in Croatia, as well as Krenica Lake in Bosnia and Herzegovina.

### Determination Key to *Cobitis* Species in Dalmatia and Herzegovina

**1a** The number of branched fin rays in the anal fin 6^1^/_2_ (exceptionally 7^1^/_2_) and in the caudal fin 15 (exceptionally 14 and 16). The whole body covered with small, yet clearly visible scales. In the surface pigmentation layer of the caudal fin base usually one small, oblique, dark spot located in the upper half of the body sides. …….……………………………………….***Cobitis jadovaensis***

**1b** In the anal fin usually 5^1^/_2_ (exceptionally 6^1^/_2_) and in the caudal fin 14 branched rays. ………………………………….…..**2**

**2a** Two dark spots in the surface pigmentation layer of the caudal fin base. ………………………………………**3**

**2b** One dark spot or no dark spots in the surface pigmentation layer of the caudal fin base. ………………………**4**

**3a** On the caudal fin base clearly visible two dark spots (located in the surface pigmentation layer), most often oblique in shape, the upper one being larger. In Z4 usually clearly separated big squares and rectangles. In dorsal fin 6^1^/_2_–7^1^/_2_ branched rays. ………………………………………………………***C. bilineata***

**3b** Usually two dark spots in the surface pigmentation layer of the caudal fin base–the upper one darker and oblique, the lower one being lighter and roundish, even though exceptions are possible. Squares and rectangles in Z4 often partly or completely merged. In dorsal fin usually 7^1^/_2_, exceptionally 6^1^/_2_ or 8^1^/_2_ branched rays. …………………………..…….….***C. narentana***

**4a** On the caudal fin base only an upper dark spot is usually visible (belonging to the surface pigmentation layer); exceptionally another, lower and lighter, spot (belonging to the deeper pigmentation layer present. In dorsal fin 6^1^/_2_, exceptionally 5^1^/_2_ branched rays. ……………………………………………...…***C. illyrica***

**4b** No pronounced spots on the caudal fin base, rarely small hardly visible spots irregular in shape and belonging to the deeper pigmentation layer can be noticed. The number of branched dorsal fin rays 6^1^/_2_–7^1^/_2_. ..…...………………………**5**

**5a** No dark spots on the caudal fin base. The number of branched dorsal fin rays 6^1^/_2_–7^1^/_2_. Small scales on the whole body surface. ……………………………..........…***C. dalmatina***

**5b** No spots on the caudal fin base, or, rarely, small and hardly visible spots present, located in the deeper layer. The number of branched dorsal fin rays 6^1^/_2_. Scales very small, visible only under magnification. ….***C. herzegoviniensis*** **Buj & Šanda, sp. nov.**

## Supporting Information

Table S1 **Mean, minimal and maximal values of morphometric ratios of investigated** ***Cobitis*** **species.**
(XLSX)

## References

[1] Chen X, Jiang K, Guo P, Huang S, Rao D, et al. (2014) Assessing species boundaries and the phylogenetic position of the rare Szechwan ratsnake, *Euprepiophis perlaceus* (Serpentes: Colubridae), using coalescent-based methods. Mol Phylogenet Evol 70: 130–136. 10.1016/j.ympev.2013.09.003 24060366

[2] Knowles LL, Carstens BC (2007) Delimiting Species without Monophyletic Gene Trees. Syst Biol 56: 887–895. 10.1080/10635150701701091 18027282

[3] Sites JW, Marshall JC (2004) Empirical criteria for delimiting species. Ann Rev Ecol Evol Syst 35: 199–227.

[4] Carstens BC, Dewey TA (2010) Species Delimitation Using a Combined Coalescent and Information-Theoretic Approach: An Example from North American *Myotis* Bats. Syst Biol 59: 400–414. 10.1093/sysbio/syq024 PMC2885268 20547777

[5] Grummer JA, Bryson jr RW, Reeder TW (2014) Species Delimitation Using Bayes Factors: Simulations and Application to the *Sceloporus scalaris* Species Group (Squamata: Phrynosomatidae). Syst Biol 63: 119133. 10.1093/sysbio/syt069 24262383

[6] Schulter D (1996) Ecological speciation in postglacial fishes. Phil Trans R Soc Lond B 351: 807814.

[7] Taylor EB (1999) Species pairs of north temperate freshwater fishes: Evolution, taxonomy, and conservation. Rev Fish Biol Fisher 9: 299–324.

[8] Bernardi G (2013) Speciation in fishes. Mol Ecol 22: 54985502. 10.1111/mec.12494 24118417

[9] Kottelat M, Freyhof J (2007) Handbook of European freshwater fishes. Kottelat, Cornol, Switzerland and Freyhof, Berlin, Germany. 646 p.

[10] Kottelat M (2012) Conspectus Cobitidium: An inventory of the loaches of the world (Teleostei: Cypriniformes: Cobitoidei). Raffles B Zool Suppl. 26: 1–199.

[11] Mustafić P, Marčić Z, Duplić A, Mrakovčić M, Ćaleta M, et al (2008) A new loach species of the genus *Cobitis* in Croatia. Folia Zool 57: 4–9.

[12] Vasil’eva ED (2000) Sibling species in the genus *Cobitis* (Cobitidae, Pisces). Folia Zool 49: 23–30.

[13] Bickford D, Lohman DJ, Sodhi NS, Ng PKL, Meier R, et al (2006) Cryptic species as a window on diversity and conservation. Trends Ecol Evol 22: 148–155. 17129636 10.1016/j.tree.2006.11.004

[14] Schneider D, Mrakovčić M, Mustafić P, Kerovec M (2000) Morphological differences in some *Cobitis* populations from Croatia. Folia Zool 49: 227–234.

[15] Freyhof J, Stelbrink B (2007) *Cobitis illyrica*, a new species of loach from Croatia (Teleostei: Cobitidae). Ichthyol Explor Fres 18: 269–275.

[16] Ráb P, Rábová M, Bohlen J, Lusk S (2000) Genetic differentiation of the two hybrid diploid-polyploid complexes of loaches, genus *Cobitis* (Cobitidae) involving *C. taenia*, *C. elongatoides* and *C*. spp. in the Czech Republic: karyotypes and cytogenetic diversity. Folia Zool 49: 55–66.

[17] Boroń A (2003) Karyotypes and Cytogenetic Diversity of the Genus *Cobitis* (Pisces, Cobitidae) in Poland: a Review. Cytogenetic Evidence for a Hybrid Origin of some *Cobitis* Triploids. Folia Biol (Krakow) 51: 49–54. 15303340

[18] Perdices A, Doadrio I (2001) The molecular systematics and biogeography of the European cobitids based on mitochondrial DNA sequences. Mol Phylogenet Evol 19: 468–478. 11399153 10.1006/mpev.2000.0900

[19] Janko K, Vasil’ev VP, Ráb P, Rábová M, Šlechtova V, et al (2005) Genetic and morphological analyses of 50–chromosome spined loaches (*Cobitis*, Cobitidae, Pisces) from the Black Sea basin that are morphologically similar to *C. taenia*, with the description of a new species. Folia Zool 54: 405–420.

[20] Bohlen J, Perdices A, Doadrio I, Economidis PS (2006) Vicariance, colonization, and fast local speciation in Asia Minor and the Balkans as revealed from the phylogeny of spined loaches (Osteichthyes; Cobitidae). Mol Phylogenet Evol 39: 552–561. 16439160 10.1016/j.ympev.2005.12.007

[21] Buj I, Podnar M, Mrakovčić M, Choleva L, Šlechtova V, et al (2008) Genetic diversity and phylogenetic relationships of spined loaches (genus *Cobitis*) in Croatia based on mtDNA and allozyme analyses. Folia Zool 57: 71–82.

[22] Perdices A, Bohlen J, Doadrio I (2008) The molecular diversity of Adriatic spined loaches (Teleostei, Cobitidae). Mol Phylogenet Evol 46: 382–390. 17625922 10.1016/j.ympev.2007.05.007

[23] Banarescu P, Herzig-Straschil B (1998) Beitrag zur Kenntnis der *Leuciscus*-Untergattung *Telestes* Bonaparte (Pisces: Cyprinidae). Ann Nat Hist Mus Wien 100: 405–424.

[24] Bogutskaya NG, Zupančič P (1999) A re-description of *Leuciscus zrmanjae* (Karaman, 1928) and new data on the taxonomy of *Leuciscus illiricus*, *L. svallize* and *L. cephalus* (Pisces: Cyprinidae) in the West Balkans. Ann Nat Hist Mus Wien 101: 509–529.

[25] Mrakovčić M, Brigić A, Buj I, Ćaleta M, Mustafić P, et al.. (2006) Red book of freshwater fish of Croatia. Zagreb: Ministry of Culture and State Institute for Nature Protection, Republic of Croatia. 253 p.

[26] Karaman S (1928) Prilozi ihtiologiji Jugoslavije. Glasnik Skopskog naučnog društva 6: 163–164 (in Serbian and in German)..

[27] Schneider D, Mustafić P, Mrakovčić M, Mihaljević Z (2000) Some aspects of the biology of the Neretvan spined loach. Folia Zool 49: 159–165.

[28] Šanda R, Bogut I, Doadrio I, Kohout J, Perdices A, et al (2008) Distribution and taxonomic relationships of spined loaches (Cobitidae, *Cobitis*) in the River Neretva basin, Bosnia and Herzegovina. Folia Zool 57: 20–25.

[29] Elliot NG, Haskard K, Koslow JA (1995) Morphometric analysis of orange roughy (*Hoplostethus atlanticus*) off the continental slope of southern Australia. J Fish Biol 46: 202–220.

[30] Turan C (2004) Stock identification of Mediterranean horse mackerel (*Trachurus mediterraneus*) using morphometric and meristic characters. ICES J Mar Sci 61: 774–781.

[31] Klingenberg CP, Gidaszewski NA (2010) Testing and Quantifying Phylogenetic Signals and Homoplasy in Morphometric Data. Syst Biol 59: 245–261. 20525633 10.1093/sysbio/syp106

[32] Kotusz J (2000) Intra- and interpopulation morphological variability in diploid and varied-ploidy *Cobitis* from Poland. Folia Zool 49: 219–226.

[33] Gambetta L (1934) Sulla variabilita del cobite fluviale (*Cobitis taenia*) e sul rapporto numerico dei sessi. Bollettino dei Musei di Zoologia ed Anatomia Comparata della Reale Universitá di Torino 44: 297–324.

[34] Saitoh T, Aizawa H (1987) Local Differentiation within the Striata Spined Loach (the *striata* Type of *Cobitis taenia* complex). Jpn J Ichthyol 34: 334?345.

[35] Hrbek T, Stolting KN, Bardakci F, Kucuk F, Wildekamp RH, et al (2004) Plate tectonics and biogeographical patterns of the *Pseudophoxinus* (Pisces: Cypriniformes) species complex of central Anatolia, Turkey. Mol Phylogenet Evol 32: 297–308. 15186815 10.1016/j.ympev.2003.12.017

[36] Perdices A, Doadrio I, Bermingham E (2005) Evolutionary history of the synbranchid eels (Teleostei: Synbranchidae) in Central America and Caribbean islands inferred from their molecular phylogeny. Mol Phylogenet Evol 37: 460–473. 16223677 10.1016/j.ympev.2005.01.020

[37] Šlechtová V, Bohlen J, Tan HH (2007) Families of Cobitoidea (Teleostei; Cypriniformes) as revealed from nuclear genetic data and the position of the mysterious genera *Barbucca*, *Psilorhynchus*, *Serpenticobitis* and *Vaillantella* . Mol Phylogenet Evol 44: 1358–1365. 17433724 10.1016/j.ympev.2007.02.019

[38] Thompson J, Gibson T, Plewniak F, Jeanmougin F, Higgins D (1997) The CLUSTAL X windows interface: flexible strategies for multiple sequence alignment aided by quality analysis tools. Nucleic Acids Res 25: 4876–4882. 9396791 10.1093/nar/25.24.4876 PMC147148

[39] Stephens M, Smith NJ, Donnelly P (2001) A new statistical method for haplotype reconstruction from population data. Am J Hum Genet 68: 978–989. 11254454 10.1086/319501 PMC1275651

[40] Stephens M, Scheet P (2005) Accounting for decay of linkage disequilibrium in haplotype inference and missing data imputation. Am J Hum Genet 76: 449–462. 15700229 10.1086/428594 PMC1196397

[41] Swofford DL (2002) PAUP*: Phylogenetic Analysis Using Parsimony (*and Other Methods), Version 4 [Computer software and manual]. Sunderland, MA: Sinauer Associates.

[42] Fu YX, Li WH (1993) Statistical tests of neutrality of mutations. Genetics 133: 693–709. 8454210 10.1093/genetics/133.3.693 PMC1205353

[43] Tajima F (1989) Statistical method for testing the neutral mutation hypothesis by DNA polymorphism. Genetics 123: 585–595. 2513255 10.1093/genetics/123.3.585 PMC1203831

[44] Librado P, Rozas J (2009) DnaSP v5: A software for comprehensive analysis of DNA polymorphism data. Bioinformatics 25: 1451–1452. 19346325 10.1093/bioinformatics/btp187

[45] Hudson RR (1987) Estimating the recombination parameter of a finite population model without selection. Genet Res 50: 245–250. 3443297 10.1017/s0016672300023776

[46] Hudson RR, Kaplan NL (1985) Statistical properties of the number of recombination events in the history of a sample of DNA sequences. Genetics 111: 147–164. 4029609 10.1093/genetics/111.1.147 PMC1202594

[47] Tamura K, Dudley J, Nei M, Kumar S (2007) *MEGA4*: Molecular Evolutionary Genetics Analysis (MEGA) software version 4.0. Mol Biol Evol 24: 1596–1599. 17488738 10.1093/molbev/msm092

[48] Huelsenbeck JP, Ronquist F (2001) MRBAYES: Bayesian inference of phylogenetic trees. Bioinformatics 17: 754–755. 11524383 10.1093/bioinformatics/17.8.754

[49] Posada D, Crandall KA (1998) MODELTEST: testing the model of DNA substitution. Bioinformatics 14: 817–818. 9918953 10.1093/bioinformatics/14.9.817

[50] Templeton AR, Crandall KA, Sing CF (1992) A cladistic analysis of phenotypic associations with haplotypes inferred from restriction endonuclease mapping and DNA sequence data. III. Cladogram estimation. Genetics 132: 619–633. 1385266 10.1093/genetics/132.2.619 PMC1205162

[51] Clement M, Posada D, Crandall KA (2000) TCS: a computer program to estimate gene genealogies. Mol Ecol 9: 1657–1659. 11050560 10.1046/j.1365-294x.2000.01020.x

[52] Xie WG, Lewis PO, Fan Y, Kuo L, Chen MH (2011) Improving marginal likelihood estimation for Bayesian phylogenetic model selection. Syst Biol 60: 150?160. 10.1093/sysbio/syq085 PMC3038348 21187451

[53] Drummond AJ, Rambaut A (2007) BEAST: Bayesian evolutionary analysis by sampling trees. BMC Evol Biol 7: 214. 17996036 10.1186/1471-2148-7-214 PMC2247476

[54] Rambaut A, Drummond AJ (2007) Tracer v1.4. Available from: URL http://beast.bio.ed.ac.uk/Tracer.

[55] Lartillot N, Philippe H (2006) Computing Bayes factors using thermodynamic integration. Syst Biol 55: 195–207. 10.1080/10635150500433722 16522570

[56] Drummond AJ, Suchard MA, Xie D, Rambaut A (2012) Bayesian phylogenetics with BEAUti and the BEAST 1.7. Mol Biol Evol 29: 1969–1973. 10.1093/molbev/mss075 PMC3408070 22367748

[57] Hey J, Wakeley J (1997) A coalescent estimator of the population recombination rate. Genetics 145: 833–846. 9055092 10.1093/genetics/145.3.833 PMC1207867

[58] Li WLS, Drummond AJ (2012) Model Averaging and Bayes Factor Calculation of Relaxed Molecular Clocks in Bayesian Phylogenetics. Mol Biol Evol 29: 751–761. 10.1093/molbev/msr232 PMC3258040 21940644

[59] Perea S, Bohme M, Zupancic P, Freyhof J, Sanda R, et al.. (2010) Phylogenetic relationships and biogeographical patterns in Circum-Mediterranean Subfamily Leuciscinae (Teleostei, Cyprinidae) inferred from both mitochondrial and nuclear data. BMC Evol Biol, doi:10.1186/1471–2148–10–265. 10.1186/1471-2148-10-265 PMC2940817 20807419

[60] Carstens BC, Knowles LL (2007) Shifting distributions and speciation: species divergence during rapid climate change. Mol Ecol 16: 619–627. 17257117 10.1111/j.1365-294X.2006.03167.x

[61] Gamble T, Berendzen PB, Shaffer HB, Starkey DE, Simons AM (2008) Species limits and phylogeography of North American cricket frogs (Acris: Hylidae). Mol Phylogenet Evol 48: 112–125. 18462953 10.1016/j.ympev.2008.03.015

[62] Marková S, Šanda R, Crivelli A, Shumka S, Wilson IF, et al (2010) Nuclear and mitochondrial DNA sequence data reveal the evolutionary history of *Barbus* (Cyprinidae) in the ancient lake systems of the Balkans. Mol Phylogenet Evol 55: 488–500. 20139017 10.1016/j.ympev.2010.01.030

[63] Buj I, Podnar M, Mrakovčić M, Ćaleta M, Mustafić P, et al (2008) Morphological and genetic diversity of *Sabanejewia balcanica* in Croatia. Folia Zool 57: 100–110.

[64] Doadrio I, Perdices A (2005) Phylogenetic relationships among the Ibero-African cobitids (*Cobitis*, Cobitidae) based on cytochrome b sequence data. Mol Phylogenet Evol 37: 484–493. 16150615 10.1016/j.ympev.2005.07.009

[65] Šanda R, Vukić J, Choleva L, Křížek J, Šedivá A, et al (2008) Distribution of loach fishes (Cobitidae, Nemacheilidae) in Albania, with genetic analysis of population of *Cobitis ohridana* . Folia Zool 57: 42–50.

